# Gold‐Stabilized Copper Enables Anodic Hydrogen Evolution for Ultralow‐Voltage CO‐to‐Ethylene Electrolysis

**DOI:** 10.1002/adma.74459

**Published:** 2026-08-09

**Authors:** Sungjin Park, Hengzhou Liu, Heejong Shin, Hafiz Ghulam Abbas, Loann Bonnenfant, Ke Xie, Hyeon Seok Lee, Fatemeh Arabyarmohammadi, Jianan Erick Huang, Panos Papangelakis, Peiying Wang, Ali Shayesteh Zeraati, Jiheon Kim, Roham Dorakhan, David Sinton, Xiaopeng Liu, Wenzhen Li, Byoung‐hoon Lee, Edward H. Sargent

**Affiliations:** ^1^ Department of Chemistry Northwestern University Evanston Illinois USA; ^2^ Department of Electrical and Computer Engineering Northwestern University Evanston Illinois USA; ^3^ Department of Electrical and Computer Engineering University of Toronto Toronto Ontario Canada; ^4^ Department of Mechanical and Industrial Engineering University of Toronto Toronto Ontario Canada; ^5^ Department of Chemical and Biological Engineering Iowa State University Ames USA; ^6^ KU‐KIST Graduate School of Converging Science and Technology Korea University Seoul Republic of Korea

**Keywords:** catalysis, copper oxide, electrochemistry, electrolysis, ethylene, furfural, hydroxide, oxygen evolution, Anodic hydrogen evolution, Carbon monoxide reduction

## Abstract

Electrochemical upgrading from CO_2_ and CO to ethylene has typically been coupled with the oxygen evolution reaction (OER), whose high standard reduction potential leads to full‐cell voltages above 2.2 V at 200 mA/cm^2^. Here we explored an alternative anodic reaction, where furfural is oxidized to furoic acid, a reaction having a low onset potential (*E* ^0^ ≈ 0.05 V vs. RHE), and which reaction is accompanied by the evolution of H_2_: an anodic hydrogen evolution reaction (a‐HER). In early experiments, copper oxide as a‐HER catalyst exhibit limited stability (< 10 min) and activity (80 mA/cm^2^ at 0.8 *V*_cell_). We found, using operando spectroscopy, that hydroxide forms on the surface of copper and deactivates the desired a‐HER process. When we screened candidate metal dopants, we found the best to be Au, for it served to stabilize the Cu surface, enablinained a‐HER: 260 mA cm^−2^ at 0.8 *V*_cell_ and stable operation for 16 h. Integrated into a paired CO‐to‐ethylene electrolyzer, this delivered 0.92 V_fullcell_ at 400 mA cm^−2^, required 40 GJ electricity per ton of ethylene, and co‐produced 460 kg H_2_ per ton ethylene. To enable comparison with CO_2_‐to‐ethylene reports, which require an additional CO_2_‐to‐CO step, we estimate ∼ 69 GJ/tonC_2_H_4_.

## Graphical abstract

Gold‐stabilized copper suppresses hydroxide‐driven deactivation during anodic hydrogen evolution from furfural. Pairing this low‐potential anode with CO‐to‐ethylene electroreduction enables sub‐1 V operation at 400 mA cm^−2^, high single‐pass CO conversion, and co‐production of ethylene, furoic acid, and a separate hydrogen stream.

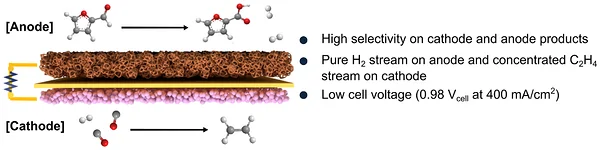

## 1 Introduction

Electrochemical carbon upgrade from CO_2_ and CO offers pathways to low‐carbon‐intensity fuels and platform chemicals. Carbon monoxide electroreduction (COR) avoids carbonate salt precipitation, allowing for stable electrolysis. Recent efforts in catalyst design and cell engineering have improved current density, faradaic efficiency (FE), and single‐pass conversion efficiency (SPCE) [1, 2, 3, 4, 5]. Nevertheless, producing ethylene electrochemically still demands 110–170 GJ per ton [6, 7, 8, 9].

This arises in part from pairing with the oxygen evolution reaction (OER), with its onset potential of 1.23 V_RHE_ (Figure 1b). Previous studies have explored pairing CO_2_R/COR at the cathode with the oxidation of organic molecules at the anode, the aim to lower the full cell voltage while producing value‐added products. However, even with these approaches, systems that involve pairing with organic oxidation reactions have yet to unite (Table S1) [10, 11] sub‐1 V operation with high current density, ethylene selectivity, and feedstock utilization (SPCE, single‐pass conversion efficiency of the feedstock).

**FIGURE 1:**
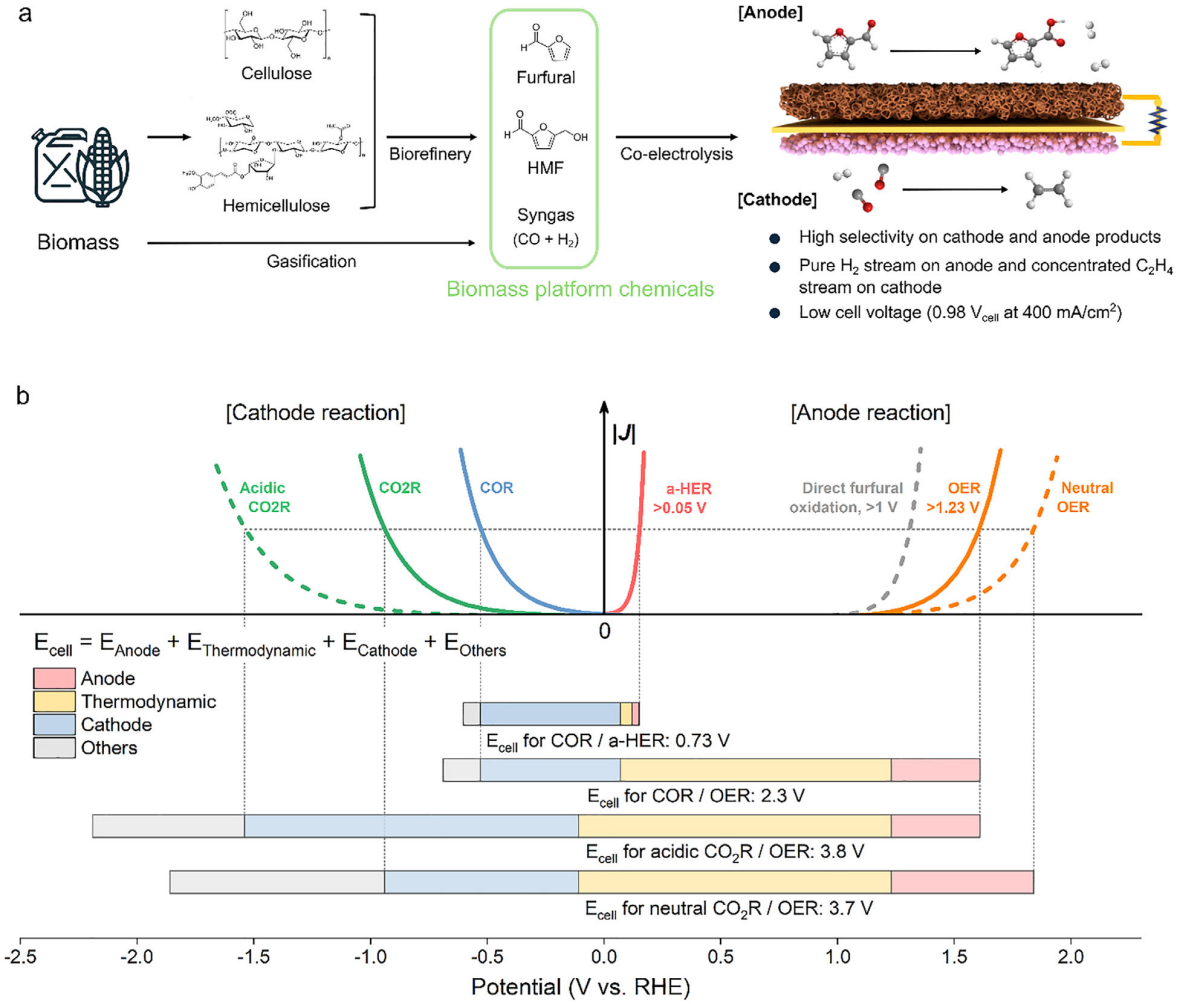
Schematic representation of the COR‐a HER system studied herein. (a) Electrocatalytic biomass upgrading toward chemicals and fuels. (b) iR‐uncorrected voltage breakdown analysis of representative cathodic CO_2_R/COR reactions paired with candidate anodic reactions at 200 mA cm^−2^ in a membrane electrode assembly (MEA) configuration. The full‐cell voltage is decomposed into anode, thermodynamic, cathode, and other contributions. Detailed calculation methods and assumptions are provided in Note S1.

Anodic hydrogen evolution (a‐HER) is an anodic alternative, one that offers a similarly lowered standard potential accompanied by the production of H_2_ at the anode outlet [12, 13, 14, 15]. In this approach, non‐enolizable aldehydes serve as both proton and electron donors, enabling electrocatalytic oxidative dehydrogenation on Cu‐based anodes at potentials of ∼0.05 V versus RHE accompanied by the evolution of H_2_. Among potential aldehyde substrates, furfural is an attractive candidate because it can be derived from lignocellulosic feedstocks such as corn stover, thereby incorporating biogenic carbon into the paired electrolysis scheme (Figure 1a).

Previous studies have explored a‐HER from furfural on copper anodes—including those coupled with cathodic hydrogen evolution (c‐HER)—but these showed < 10 min operating lifetimes at > 400 mA/cm^2^ current density [12, 13, 14, 15]. We sought here to develop anode catalyst materials that sustain efficient a‐HER at low overpotential and high current density (> 200 mA/cm^2^).

We find that the deactivation of the copper anodic catalyst arises from the facile adsorption of hydroxide ions, which promotes unwanted oxygen evolution (Figure 2d) [16, 17]. We explore a doping strategy to stabilize the oxidation state of copper (Cu^0^ and Cu^+^), thus preventing hydroxide occupation and suppressing the OER pathway. Among the dopants screened, gold demonstrates the best performance in a‐HER, inhibiting hydroxide binding at the active sites. Au‐modified oxide‐derived copper foam (AuCu) exhibits stable a‐HER performance (> 8 h, Figure 2d); and it reduces the cell voltage by 0.24 V at 200 mA/cm^2^ compared to OD‐Cu catalyst.

**FIGURE 2:**
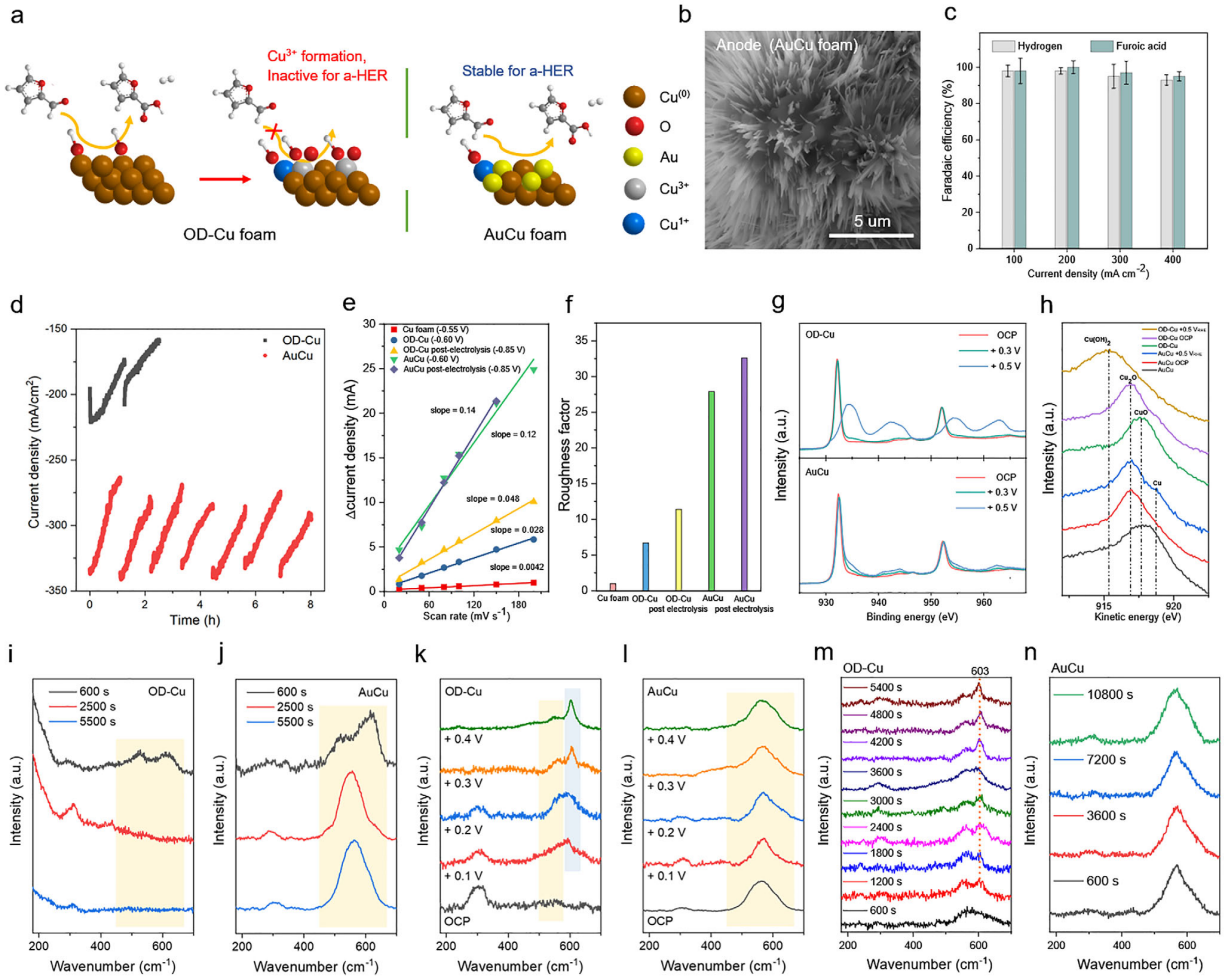
Performance and characterization of a‐HER. (a) Schematic illustration of OD‐Cu deactivation through surface Cu–O(H) formation during a‐HER and the stabilization effect of Au modification. (b) Scanning electron microscopy (SEM) image of post‐electrolysis AuCu foam. (c) Anode product distribution for a‐HER over AuCu foam at 100, 200, 300, and 400 mA cm^−2^, with the corresponding full‐cell voltages of 0.62, 0.73, 0.84, and 0.92 V, respectively. (d) Stability assessment of OD‐Cu and AuCu catalysts for the anodic hydrogen evolution reaction (a‐HER) in an H‐cell configuration at a potential of 0.5 V_RHE_, conducted in a solution containing 250 mM furfural and 1 M KOH. Note that current density was not recovered even after replenishing furfural in the anolyte for OD‐Cu. However, AuCu performed stable operation with anolyte refreshment. (e) Capacitive current plotted against the scan rate, measured in 1 M KOH, with scan rates ranging from 20 to 200 mV/s around the open circuit potential. (f) Roughness‐factor analysis of various catalysts utilized for the a‐HER. (g) High‐resolution Cu 2p XPS spectra of OD‐Cu and AuCu after electrochemical conditioning at OCP, 0.3 V_RHE_, and 0.5 V_RHE_. The Cu 2p_3/2_ features at 932.6, 932.2, 933.8, and 934.7 eV are assigned to metallic Cu, Cu_2_O/Cu^+^, CuO/Cu^2^^+^, and Cu(OH)_2_/Cu^2^^+^ species, respectively. (h) Cu LMM Auger spectra of AuCu and OD‐Cu foam measured at pristine, OCP, and 0.5 V_RHE. The Cu LMM features are assigned to metallic Cu, Cu_2_O/Cu^+^, CuO/Cu^2^^+^, and Cu(OH)_2_/Cu^2+^ contributions. Detailed deconvolution, fitting parameters, and complementary of Cu 2p3/2 and LMM Auger spectra are provided in Note S2. Operando Raman spectroscopy under reducing potential at −0.1 V_RHE_ of (i) OD‐Cu foam and (j) AuCu foam. Operando Raman spectroscopy with a‐HER reaction from 0.1 to 0.4 V_RHE_ of (k) OD‐Cu foam and (l) AuCu foam using 0.1 M KOH and 50 mM furfural. The shaded Raman region highlights Cu–O(H)‐related features formed during anodic operation. Time‐resolved operando Raman spectra of (m) OD‐Cu and (n) AuCu recorded at 0.2 V_RHE_ under conditions of 0.1 M KOH with the addition of 50 mM furfural.

To tackle system‐level issues, we sought to develop a cathode catalyst that maintains high conversion at low CO partial pressure (targeting 20 vol%, relevant to biogenic syngas) while maximizing SPCE. By developing carbon‐based supports exhibiting tailored porosity and gas transport properties, we SPCE of 90% and CO conversion to ethylene using as feedstock CO at 20vol%, and kept H_2_ FE on the cathode below 9% under these conditions. When we pair this with the AuCu anode and carry out furfural oxidation, the co‐electrolysis system delivers 63% ethylene FE at 400 mA/cm^2^, 0.92 *V*_cell_, and 93% furfural SPCE under stable 20‐h operation. The energy consumption in this system is lowered to 40 GJ/ton C_2_H_4_ compared to COR–OER benchmark systems that today remain above 100 GJ/ton C_2_H_4_.

## 2 Results and Discussion

We began our investigations with high‐surface‐area oxide‐derived copper foam (OD‐Cu) (Figure 2a; Figures S1 and S2) to explore the a‐HER using furfural as the reactant. Compared to pristine copper foam, the OD‐Cu catalyst demonstrated enhanced a‐HER activity, as seen in increased reaction rates (Figure 2d) and greater furoic acid production (Figure S1b). The increased roughness factor, as shown by the capacitive current versus scan rate plot, corresponds to a larger electrochemically active surface area for OD‐Cu (Figures 2e,f and S3). When applied to a membrane electrode assembly (MEA) cell, OD‐Cu delivered a current density of 300 mA/cm^2^ at 1.1 *V*_cell_ (Figure 4c). Unfortunately, this class of catalyst suffered performance decline after operating for 10 min at 300 mA/cm^2^ (Figures 2d and 4d).

Prior studies have shown that such hydroxide‐rich surface layers readily form on copper electrodes in alkaline media, particularly under anodic potentials [16, 17, 18, 19, 20]. These adsorbed hydroxide layers can block active sites and hinder the adsorption of organic molecules such as furfural [13]. X‐ray photoelectron spectroscopy (XPS) of OD‐Cu at open circuit potential (OCP), 0.3 and 0.5 V_RHE_ revealed surface oxidation to Cu(OH)_2_ and CuO, indicated by peaks at 933.76 and 934.67 eV [21] (Figure 2g and details in Note S2). Strong satellite peaks of Cu(II) at 0.5 V_RHE_, along with a Cu LMM peak at 916.5 eV (Cu(OH)_2_), indicate hydroxide adsorption onto the copper surface (Figure 2h). Operando x‐ray absorption spectroscopy (XAS) of OD‐Cu showed a predominance of Cu^2^^+^ species, suggesting Cu(OH)_2_ formation on the surface (details in Note S3).

Guided by prior work showing that metal dopants shift the oxidation of Cu [22, 23, 24], we incorporated noble metals (Au, Pt) into OD‐Cu scanning electron microscopy (SEM), transmission electron microscopy (TEM), energy‐dispersive x‐ray spectroscopy (EDX), and x‐ray diffraction analysis (XRD) indicate metal incorporation into OD‐Cu (Figures 2b,g,h and S4–S8). Additional analyses including grazing incidence x‐ray diffraction (GI‐XRD), SEM‐EDX, TEM‐EDX, Au 4f XPS, and Au L_3_‐edge XAS indicate that Au is incorporated principally as surface‐deposited Au‐containing domains with local Au‐Cu interfacial motifs, rather than through homogeneous bulk alloying or atomically‐dispersed Au (Note S2). This assignment is consistent with surface‐limited galvanic replacement/deposition during HAuCl_4_ treatment, in which AuCl_4_^−^ is reduced by surface Cu/Cu+ species without a high‐temperature alloying step. Both Au‐ and Pt‐modified Cu (AuCu and PtCu) showed improved catalytic activity and stability (Figures 2c–f and 4c,d). AuCu provided the lowest overpotential for a‐HER (Figure 4c). We found a Cu:Au ratio of 10:1 to be optimal: it provided the lowest overpotential and selectively produced furoic acid and hydrogen (Figure S9).

We employed operando and post‐electrolysis spectroscopy to monitor the chemical state of Cu on OD‐Cu and AuCu surfaces. Post‐electrolysis XPS showed that AuCu combined metallic copper (Cu^0^, 932.6 eV) [21] and copper in the (I) oxidation state (Cu_2_O, 932.4 eV) [21] at OCP, 0.3 V_RHE_, and 0.5 V_RHE_. In contrast, OD‐Cu evolved toward the copper (I) state characteristic of hydroxide (Cu(OH)_2_, 934.7 eV) [21] at 0.5 V_RHE_ (Figure 2g). Because Cu^0^ and Cu^+^ are difficult to distinguish using Cu 2p spectra alone, we analyzed Cu LMM Auger spectra (Figure 2h). From Cu 2p and Cu LMM fitting, OD‐Cu becomes dominated by Cu^2+^ oxide/hydroxide species after applying a potential +0.5 V_RHE_; whereas AuCu retains a larger low‐valent Cu fraction (either Cu^0^ or Cu^+^) under the same condition. The Cu^2^^+^ contribution at +0.5 V_RHE_ is higher for Cu than for AuCu in both Cu 2p and Cu LMM analyses. Thus the addition of Au suppresses Cu hydroxylation/oxidation and stabilizes low‐valent Cu species under a‐HER‐relevant anodic potentials. Detailed peak assignments, fitting constraints, and quantitative fitting results are provided in Note S2.

Operando Raman spectroscopy indicated the stable coexistence of Cu^0^ and Cu^+^ species in AuCu over a wide potential range (−0.1 to 0.5 V_RHE_), including a‐HER conditions (Figure 2j,l). A persistent broad band centered at ∼550 cm^−1^ indicates the presence of disordered or amorphous Cu^+^‐O domains, likely involving short‐range Cu^+^─O bonding stabilized by Au incorporation [25, 26]. This agrees with prior operando Raman studies, which associate abroad signal around ∼550 cm^−1^ with a defect‐rich, mixed‐valence Cu oxide network [27]. The chemical stability of AuCu across both reducing and oxidizing potentials (–0.1 to 0.5 V_RHE_) can be attributed to the electronic influence of gold: interfacial Au withdraws electron density from neighboring Cu atoms, generating partially cationic Cu (Cu^δ+^) sites that bind oxygen more strongly and stabilize Cu‐O coordination [28, 29, 30, 31, 32]. Time‐dependent operando Raman spectroscopy of AuCu indicates stable Cu‐O coordination over 3 h, consistent with the stable a‐HER performance of the AuCu catalyst (Figure 2n). At a mild reducing potential (–0.1 V_RHE_), OD‐Cu exhibited complete reduction to metallic copper (peaks at 523 and 623 cm^−^^1^ disappearing within 30 min, Figure 2i), indicating a greater tendency for changes in Cu–O coordination under applied bias. These Raman observations, together with XPS and in situ XAS data (Figure S32 and details in Note S3), indicate that Au stabilizes a mixed Cu^0^/Cu^+^ state over a broad potential window, whereas OD‐Cu readily undergoes redox transitions depending on the applied potential.

We applied anodic (oxidative) potentials to OD‐Cu to trigger a‐HER, upon which we measured hydrogen at the anode. Starting at 0.2 V_RHE_, a Raman peaks at 548 and 603 cm^−^^1^ emerged and intensified with increasing anodic potential (Figure 2k). These Raman features are specific to OD‐Cu, as shown by peak deconvolution of Raman spectra at 0.4 V_RHE_ (Figures S29–S31 and details in Note S3), and the Raman features became more pronounced over time (Figure 2m). Previous studies have identified the 548 and 603 cm^−^^1^ Raman peaks as Cu‐OH vibrations, formed via hydroxide adsorption onto copper surfaces [16, 17]. We propose that the strong hydroxide adsorption and the subsequent formation of copper hydroxide layer on OD‐Cu surface blocks furfural access to active sites, suppressing a‐HER (Figure 2a,d; Figures S37 and S38). OD‐Cu showed limited stability (<10 min) in both H‐cell and MEA configurations, whereas AuCu maintained stable performance for 20 h at 0.8 *V*_cell_ (Figures 2d and 4c,i; details in Note S8).

To investigate further the hydroxide‐adsorbing behavior of OD‐Cu, we examined (i) OH adsorption across different charge states and (ii) water interface interactions [33, 34]. DFT suggests that hydroxide adsorption becomes favorable on OD‐Cu as the surface charge density increases (Figure 3a). We tracked, over a 10 ps trajectory, both structural evolution and Cu─O bond length fluctuations (Figures S34, S35, and S38) to evaluate the stability of hydroxyl interactions. At higher surface charge and in alkaline media, OH‐ adsorption on OD‐Cu appeared more favorable (Figure 3b), whereas the AuCu surface showed weaker water interactions with longer Cu─O bond lengths (2.1–2.4 Å), which may hinder surface hydroxylation (Figures S34 and S35).

**FIGURE 3:**
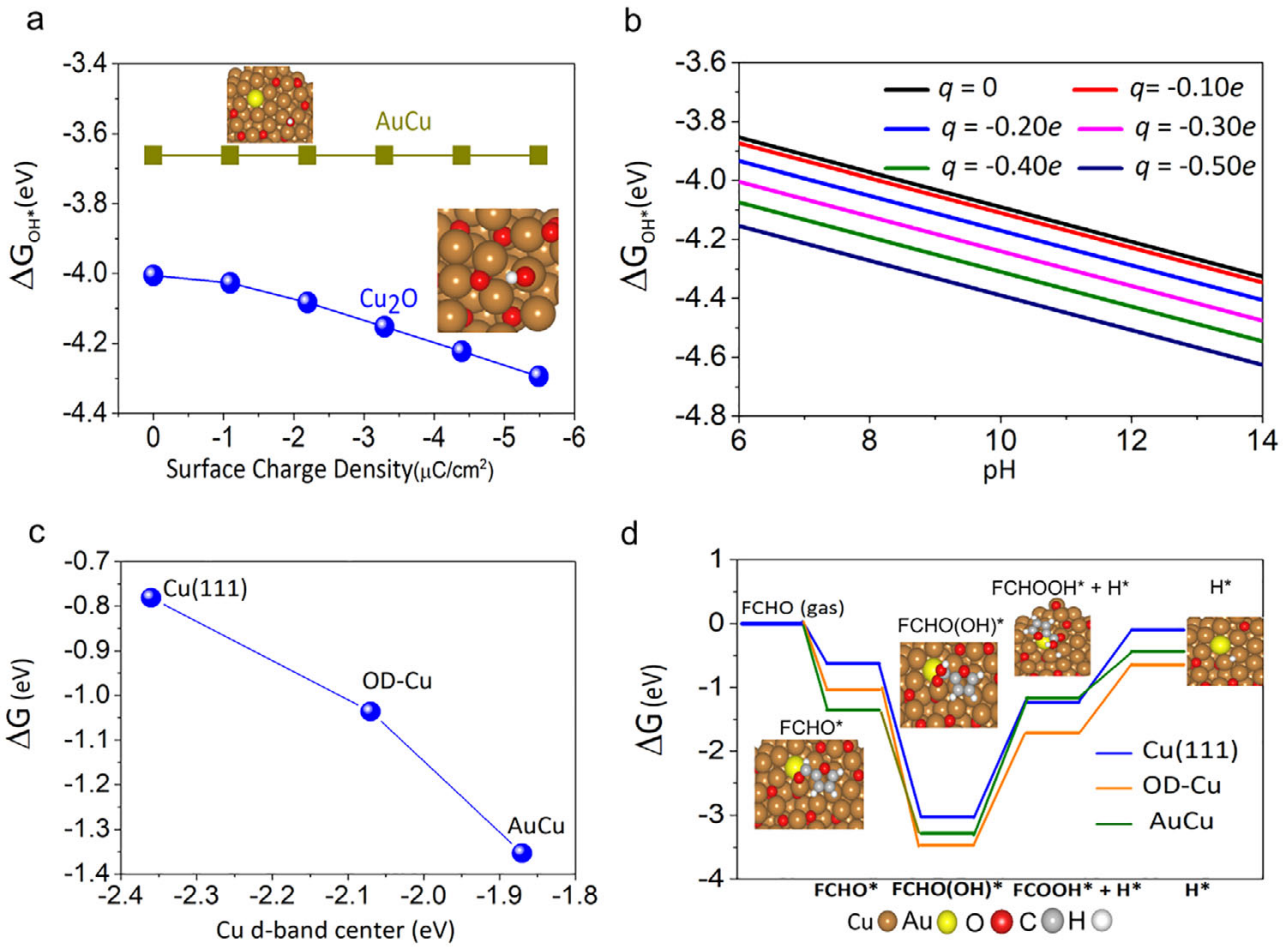
Computational studies of the furfural oxidation reaction. (a) Relationship between the adsorption energy (Δ*G*) of adsorbed hydroxyl species (OH)_ads_ and surface charge density on AuCu and Cu_2_O(111) surface. The inset image shows the most stable OH* adsorption configurations on AuCu and Cu_2_O(111). (b) The adsorption energy (Δ*G*) of (OH)_ads_ on Cu_2_O(111) as a function of pH under different charge states, providing insights into the role of electrochemical conditions in adsorption energetics. (c) Correlation between the adsorption energy (Δ*G*) of furfural on Cu(111), OD‐Cu, and AuCu with the d‐band center of surface Cu atoms. (d) Adsorption energy (Δ*G*) profile of the furfural oxidation reaction (FFOR) on Cu (111), OD‐Cu, and AuCu. The inset depicts the most stable adsorption configurations of key intermediates on the most active AuCu surface for FFOR (refer to Supporting Information for additional intermediate structures). The yellow, brown, grey, red, and white spheres represent Au, Cu, C, O, and H atoms, respectively.

We performed non‐spin‐polarized density functional theory (DFT) calculations to assess furfural adsorption energies (∆G) across Cu(111), OD‐Cu, AuCu, and hydroxylated Cu (Cu(OH)ads) (Figures S29–S31). The calculated adsorption strength followed the trend: AuCu > OD‐Cu > Cu(111) > Cu(OH)_ads_. These calculations are consistent with operando observations: OD‐Cu's tendency to form hydroxylated Cu species is associated with weaker furfural adsorption (details in Note S4), which favors OER pathway, while AuCu's moderated surface environment correlates with stronger furfural adsorption and greater a‐HER activity. Furfural adsorption was strongest on the AuCu (∆G = −1.35 eV), which may be attributed to an upward shift in d‐band center induced by Au substitution (Figures 3c and S39). This enhanced interaction stabilized furfural and facilitated key steps in the oxidation process (in this case, anodic hydrogen evolution reaction). The calculated ∆G for the rate‐determining step‐furoic acid desorption‐was lowest for AuCu (0.81 eV), compared to OD‐Cu (1.06 eV) and Cu(111) (1.13 eV) (Figure 3d). In contrast, Cu(OH)_ads_ showed weak furfural adsorption (∆G = −0.05 eV), as active sites were blocked by OH groups. These findings provide key insights into the catalytic behavior of both systems: OD‐Cu exhibits high activity toward the oxygen evolution reaction (OER), while multi‐valent AuCu preferentially facilitates a‐HER. By integrating Δ*G* trends, electronic structure analysis, and stability investigations, our calculation links the d‐band center shift, adsorption strength, and catalytic performance. The electronic modulation induced by Au substitution enhances furfural adsorption and also facilitates key reaction steps.

Taken together, these results suggest that Au doping stabilizes a mixed Cu^0^/Cu^+^ state over a broad potential window, whereas OD‐Cu readily undergoes redox transitions depending on the applied potential. Specifically, under the a‐HER potential range of 0.1–0.5 V_RHE_, OD‐Cu more readily forms copper hydroxides, leading to deactivation of the a‐HER pathway, whereas AuCu maintains stable Cu oxidation states and preserves catalytic activity.

We then turned our focus to developing a co‐electrolysis system coupling cathodic COR and anodic HER, focusing on high single‐pass CO conversion efficiency (SPCE) and efficient CO utilization even under modest CO partial pressures (20%–45%), conditions representative of biomass‐derived syngas (Figure 1a). In membrane electrode assembly (MEA) configurations, the local environment at the catalyst surface differs from that in liquid‐electrolyte flow cells, as most of the proton source exists in vapor form, requiring efficient gas transport near the catalyst interface to sustain high selectivity and reaction rates [35, 36, 37]. Previous reports suggest that introducing porous carbon nanoparticles into electrode can enhance mass transport by forming local reactant reservoirs, thereby increasing CO availability adjacent to active sites [7, 38].

We first screened a series of copper catalysts—sputtered Cu, passivated Cu nanoparticles, Cu_2_O, and CuO—for their performance toward ethylene formation in CO electroreduction (COR) (Figure S14). Among these, CuO exhibited the highest ethylene selectivity and was selected for further study. We then explored several porous carbon supports (C‐supports) as potential CO reservoirs to enhance local CO availability near active sites (Figures 4e–g and S12–S16; Table S4). All CuO/C‐support electrodes that we studied—including the control (bare CuO)—showed enhanced ethylene selectivity under reduced CO feed rates (Figure 4e; Figures S15 and S16). However, the maximum attainable ethylene FE and SPCE varied with the choice of carbon support, suggesting a correlation with their intrinsic gas‐transport properties (Figure S16). Brunauer–Emmett–Teller (BET) analysis showed that C‐support_3 had the highest surface area (725 m^2^/g) among candidates (Figures 4b and S10a). Incorporating C‐support_3 into CuO electrodes and optimizing the catalyst‐to‐ionomer ratio led to a peak ethylene FE of 63% and an SPCE of 93% at 400 mA cm^−^^2^ (Figures S12 and S13).

**FIGURE 4:**
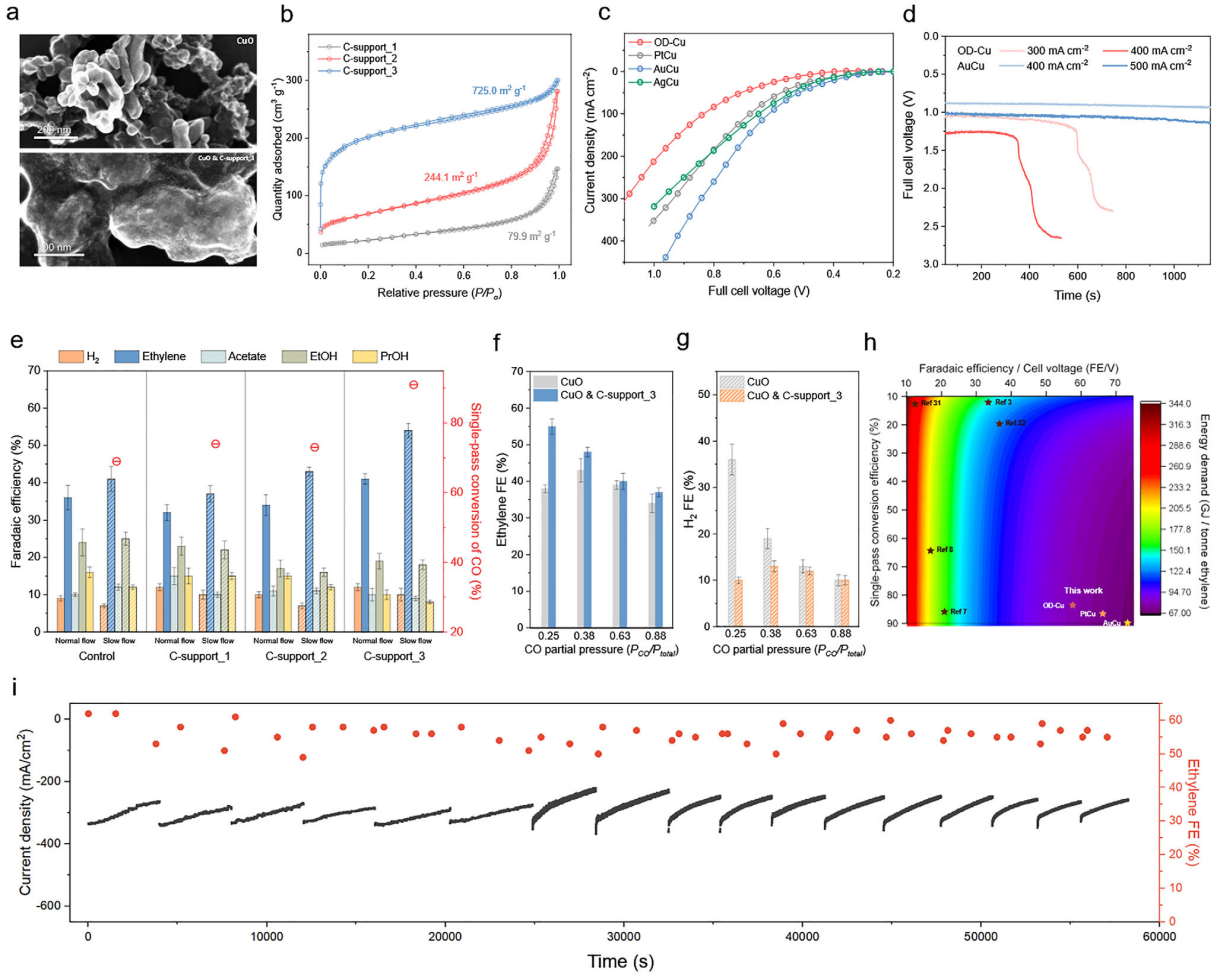
Enhanced co‐electrolysis performance via CuO/C‐supports coupling. (a) Transmission electron microscopy (TEM) image of CuO and C‐support_3 and CuO from post‐electrolysis cathode. (b) N_2_ adsorption‐desorption isotherms over different C‐supports. (c) Linear sweep voltammetry (LSV) curve using OD‐Cu, PtCu, AgCu, and AuCu catalyst for cathodic COR and anodic HER configuration without iR compensation and (d) Short‐term stability test of COR‐a HER system using OD‐Cu foam and AuCu foam. (e) Comparison of product distribution at 200 mA/cm^2^ and SPCE for various CuO and C‐supports under limited CO flow and (f) ethylene FE and (g) hydrogen FE of CuO and CuO; and C‐support_3 under simulated syngas reduction. (h) Heat map illustrates the relationship between energy demand for ethylene production, FE/V, and SPCE values. (i) Long‐term stability of the optimized catalyst operated at *V*_cell_ = 0.8 V in a membrane electrode assembly (MEA). The previous explanation that the current decay originates from furfural depletion and is fully restored after furfural replenishment should be replaced. Detailed component‐level stability diagnostics are provided in Note S8.

We used electrical impedance spectroscopy (EIS, Figure S10b) to study C‐support_3, finding that it had the highest relaxation time constant (τ_0_) and static capacitance among supports studied, consistent with prolonged local CO residence time and a larger electrochemically accessible surface area [39, 40, 41, 42, 43, 44, 45]. Together with TEM images showing uniform dispersion of Cu catalyst, ionomer, and C‐support_3 (Figures 4a and S8), and DFT results identifying the CuO/C interface as a CO‐stabilizing molecular reservoir (Figure S33), we offer that C‐support_3 sustains high local CO availability under CO‐limited conditions. Decreasing the CO partial pressure from 1 to 0.25 atm increased the ethylene FE to 55% for CuO–C‐support_3, whereas CuO alone showed dominant HER below 0.38 atm CO at 200 mA cm^−^^2^ (Figure 4f,g). This agrees with recent studies showing that excess CO coverage can saturate the Cu surface and shift the rate‐determining step away from C–C coupling toward hydrogenation of CO‐derived intermediates [45, 46, 47]. Similar trends with H_2_ dilution (Figure S16) highlight the role of carbon supports with high CO retention and accessible surface area in maximizing ethylene selectivity. Among the alkali cations tested, 1 M LiOH delivered the highest ethylene FE of 55% at 200 mA cm^−^^2^ under normal CO flow (Figure S17).

When we paired the cathodic COR reaction with the a‐HER reported above, the system had an ethylene FE of 63% together with an SPCE of 93% at a full‐cell voltage of 0.92 V and a current density of 400 mA/cm^2^. The system provided stable performance for over 20 h at 0.8 *V*_cell_ (Figure 4i). Extended stability diagnostics showed that furfural‐based operation was limited mainly by furan‐derived membrane/cell fouling, and not by AuCu anode deactivation. To further evaluate AuCu anode stability while minimizing this fouling pathway, we performed a formaldehyde‐based a‐HER control test, which operated for 75 h with sustained anode H_2_ and formate FE (Note S8).

One recent assessment of ethylene from electrochemical carbon upgrading has set a goal for OER‐coupled ethylene production of 80 GJ per ton ethylene [7]. The energy cost of the COR‐a‐HER system is 69 GJ per ton ethylene (details Note S5), enabled by its low cell voltage; we compare this with prior studies pairing COR/CO_2_R with the oxidation of other organic molecules in Figure 4h and Table S1. We estimated the plant‐gate levelized cost per ton of ethylene (details in Note S6), including separation costs estimated by ASPEN simulation (Note S7) and including the cost of consumed KOH and H_2_SO_4_ for regeneration of furoic acid; and found this total estimated cost to be less than the sum of hydrogen, ethylene, propanol, and ethanol product values (Figure S41).

## 3 Conclusion

Toward the goal of lowering cell potential for COR and improving the technoecononics of low‐carbon‐intensity C_2_H_4_, we studied pairing with a‐HER of furfural to 2‐furoic acid. OD‐Cu is prone to deactivation during a‐HER, forming copper hydroxide species on its surface, as observed using operando Raman and x‐ray spectroscopy. Au preserves the desired mixed Cu oxidation states at the applied oxidative potential, resulting in improved stability at 400 mA/cm^2^. This enabled us to build a paired electrolysis system having SPCE of 93%, ethylene FE of 63%, and 69 GJ per tonne ethylene accompanied by generation of a substantially pure, separate, H_2_ stream on the anode.

## 4 Experimental Section

### 4.1 Materials and Chemicals

Copper (II) oxide (CuO), ammonium persulfate ([NH_4_]_2_S_2_O_8_), chloroauric acid (HAuCl_4_), and chloroplatinic acid (HPtCl_4_) were purchased from Sigma‐Aldrich. Potassium hydroxide (KOH) and sulfuric acid (H_2_SO_4_) were purchased from Caledon Laboratory Chemical. The gas‐diffusion layer (Freundenberg H15C13) was purchased from Fuel Cell store. Anion‐exchange membrane (Sustainion X37‐50 Grade RT) were purchased from Dioxide Materials. Titanium mesh was received from Fuel Cell Store. The anion‐exchange membranes were activated before use [33]. The Vulcan XC 72R, Super P conductive and Carbon black, acetylene, 50% compressed were purchased from Sigma‐Aldrich. The activated carbon from Bamboo leaves was received from US Research Nanomaterial. D10 carbon particle was received from Norit. Copper foam was purchased from Aliexpress. The deionized water was prepared with a resistivity of 18.2 MΩcm.

### 4.2 Electrode Preparation

To prepare the cathode, copper(II) oxide and carbon particles (weight ratio 10:1) were dispersed in a mixture of ethanol and Nafion perfluorinated resin solution (e.g., for 40 mg of copper(II) oxide, 110 µL of Nafion solution was added) (5 wt% in a mixture of lower aliphatic alcohols and water; Sigma‐Aldrich). The dispersion was ultrasonicated for 4 h to ensure a homogeneous ink suitable for spray coating. The resulting ink was then spray‐coated onto Freudenberg carbon paper (H15C13) with a catalyst loading of 1 mg/cm^2^.

To synthesize oxide‐derived Cu foam, Cu foam samples were initially immersed in a 0.5 M H_2_SO_4_ solution for 5 min to eliminate surface impurities, followed by thorough rinsing with water. Subsequently, the rinsed Cu foam underwent agitation in a solution composed of 50 mL of 2.5 M KOH and 0.2 M (NH_4_)S_2_O_8_ for a duration of 20 min. The resulting Cu(OH)_2_/Cu foam was then washed with water and subjected to drying at 80°C in an oven. Following the drying process, the Cu(OH)_2_ foam was transferred to a CVD furnace, where argon gas (flow rate: 50–100sccm) was employed to gradually increase the temperature to 550°C over a span of 2 h, followed by a 2‐h hold at 550°C. For the surface decoration with platinum and gold, 5 mL of 50 mM HPtCl_4_ and HAuCl_4_ solutions, respectively, were evenly dispersed onto the synthesized OD‐Cu foam. The foams were then sufficiently rinsed with deionized water and dried overnight at 80°C in an oven. The synthesized foams (OD‐Cu, AuCu, and PtCu) installed at the working electrode were reduced at −0.1 V_RHE_ under 1 M KOH, using a Ag/AgCl electrode as the reference electrode and Pt foil as the counter electrode, until the polarization curve stabilized.

### 4.3 Material Characterization

SEM imaging was conducted using a Hitachi FE‐SEM SU5000 microscope. High‐angle annular dark‐field scanning transmission electron microscopy (HAADF‐STEM) imaging and EDX elemental mapping were performed with a Hitachi HF‐3300 microscope operating at 300 kV. Cathode structure and surface composition analysis were carried out using XPS (model 5600, PerkinElmer) with a monochromatic aluminum x‐ray source, respectively. X‐ray diffraction (XRD) analysis was performed on a MiniFlex600 system employing Cu Kα radiation.

Operando Raman measurements were carried out employing a Renishaw Raman Microscope equipped with a water immersion objective (×63) and a 785 nm laser. The measurements were conducted in a modified flow cell containing a 1 M KOH aqueous solution as the electrolyte. Different prepared cathode catalysts, an Ag/AgCl reference electrode (3 M KCl, BASi), and a platinum coil served as the working electrode, reference electrode, and counter electrode, respectively. CO or Ar gas was continuously supplied to the chamber during the measurements. The potential (E) recorded in the Raman spectroscopy were converted to values versus the reversible hydrogen electrode (RHE) using the equation: *E*_RHE_ = *E*_Ag/AgCl_ + 0.210 V + 0.0591 × pH.

Operando XAS investigations were carried out at the 20‐BM beamline of the canadian light source (CLS), located at the Saskatoon Research Institute. The same experimental conditions were used as in the a‐HER in a specially designed flow‐cell reactor with a window sealed with Kapton tape. The XAS signals were collected in fluorescence‐yield mode using a Vortex detector. Cu K‐edge XAS spectra were taken in the energy range of 8800–9600 eV. The raw data were processed with the ATHENA software.

### 4.4 Electrochemical Measurements

Without specification, all the COR performance was measured in the MEA electrolyzer (SKU: 68732; Dioxide Materials) at the room temperature. During MEA electrolyzer assembly, the prepared cathode electrode (1 cm × 1 cm) was positioned on the cathode side, followed by placement of the activated Sustainion membrane (5 cm × 5 cm) and the synthesized anode electrode on the anode side, successively, within the MEA electrolyzer. The catholyte (1 M KOH) and anolyte (1 M KOH with 250 mM furfural) were circulated using peristaltic pumps at a rate of 15 mL min^−1^. The applied current was regulated using an Autolab potentiostat/galvanostat.

CO gas (Linde, 99.99%) was continuously directed to the humidifier with distilled water at varying feed rates and then supplied to the cathode chamber. For simulated syngas reduction experiments, N_2_ and CO flows were controlled and mixed to achieve the desired CO local partial pressure in the feed gas.

The performance of the cathode electrode was evaluated in a two‐electrode system at different current densities using an electrochemical station (AUT50783) equipped with a current booster (10 A). Long‐term operational testing was also conducted within the same MEA electrolyzer configuration. In stability tests of the device, the anolyte of 1 M KOH with 250 mM furfural was refreshed to ensure a consistent furfural concentration.

Linear sweep voltammetry (LSV) was performed in a three‐electrode cell and MEA at a scan rate of 5 mV s^−1^ to minimize the effect of capacitance. Cyclic voltammetry (CV) was conducted with a general three‐electrode configuration from −0.9 to −0.4 V (vs. Ag/AgCl) at scan rates of 20 to 200 mV s^−1^ in Ar‐saturated 1 M KOH electrolyte to measure the electrochemically active surface area (ECSA). Electrochemical impedance spectroscopy (EIS) was obtained in the same cell configuration as CV using an EIS instrument (VIONIC, Metrohm) under Ar‐saturated 1.0 M KOH to evaluate the mass‐transfer process in C‐supports (cathode). EIS measurements in the MEA configuration were also performed for voltage breakdown analysis following previous methods [42].

### 4.5 Product Analysis

The cathode‐side products underwent separation using a simplified cold trap to distinguish between liquid and gas products. To prevent non‐faradaic cannizzaro reactions, sulfuric acid was immediately added to neutralize the collected anode side products. Gas products were analyzed using gas chromatography (Shimadzu2014, PerkinElmer Clarus 580) equipped with a thermal conductivity detector (TCD) and a flame ionization detector (FID) with a Methanizer.

The liquid products were analyzed using an NMR spectrometer (Agilent DD2 600 MHz) with dimethylsulfoxide as an internal standard. The Faradaic efficiency (FE) of liquid products was calculated by considering the total amount of products collected from both the anode and cathode sides over the same period.

The Faradaic efficiency (FEi) for each product (i) was calculated using the equation:

$$FE_{i}=\frac{n_{i}z_{i}F}{Q}\times 100\;\%$$

where *n_i_* is the moles of product *i*; *z_i_* is the number of electrons transferred for one product molecule; *F* is the Faraday constant (96,485 C mol^−1^); *Q* is the total charge passed through the electrolytic cell.

The single‐pass conversion efficiency (SPCE) can be calculated by the equations as follows:

$$\mathrm{SPCE}=\left(1-\frac{n_{\mathrm{CO}\;\left(g\right)}}{n_{\mathrm{CO}}^{0}}\right)\times 100\%=\left(1-\frac{2Fn_{\mathrm{CO}\;\left(g\right)}}{Q}\right)\times 100\%$$

### 4.6 Techno–Economic Assessment (TEA) and Energy Demand Analysis

TEA and energy demand analysis were performed following previous method [5, 6]. For Aspen simulation, we used AspenPlus v10 (AspenTech Inc., Bedford, MA) model and design the purification process. Simulations are performed based on the gas outlet compositions of this work to assess the overall separation energy requirements to obtain a polymer grade ethylene. Detailed information such as parameters, equations, and assumptions is described in Supporting Information.

### 4.7 Computational Method

Density functional theory (DFT) calculations were performed using the Vienna Ab Initio Simulation Package (VASP) with the projector‐augmented wave (PAW) method to describe electron‐ion interactions, utilizing a frozen‐core all‐electron approach [48, 49]. To capture dispersion forces, Grimme's correction was applied to the Perdew–Burke–Ernzerhof (PBE) functional [50, 51]. Structural relaxations were performed using the conjugate gradient method, optimizing atomic positions along the Hellmann‐Feynman force direction until the residual force on each atom was below 0.001 eVÅ^−^^1^. A plane‐wave energy cutoff of 520 eV was applied to ensure numerical accuracy. Adsorption energies (Δ*G*) were calculated, including zero‐point energy and entropy corrections, for four‐layered surfaces constructed with a 5×5 supercell of Cu(111), Cu_2_O(111), OD‐Cu, AuCu, and hydroxylated Cu (Cu(OH)_ads_). Water was modeled as an implicit solvent using VASPsol++ [52, 53]. Au doping was introduced by substituting Cu atoms on the top layer of mv Cu, showing a thermodynamic preference for Au occupation at Cu^0^ sites. Periodic boundary conditions were used in the xy plane, with a 30 Å vacuum region along the *z*‐axis to eliminate spurious interactions between periodic images. A 3 × 3 × 1 *k*‐point grid was employed for Brillouin zone sampling to ensure convergence of electronic states. Further details can be found in Note S3.

## Author Contributions

**E.H.S**. and **B.‐H. L**. supervised the project. **S.P**., **B.‐H. L**., and **E.H.S**. conceived the idea. **S.P**. designed the experiments and carried out the experiments. **B.‐H. L**., **S.P**., **H.L**., **P.W**., and **H.L**. synthesized catalyst. **S.P**. and **H.S**. performed the operando spectroscopy. **S.P**. and **H.S**. contributed to the SEM, TEM, and XPS data. **L.B**. carried out ASPEN simulations. **S.P**., **H.L**., and **F.A**. performed EIS and CV measurement. **J.E.H**., **P.P**., **K.X**., **H.‐S.L**., **D.K**., **P.W**., **A.Z**., **R.D**., **D.S**., and **D.K**. assisted with discussion. **S.P**., **H.S**., and **E.H.S**. cowrote the manuscript. **S.P**., **H.S**., **H.L**., **B.‐H.L**., and **E.H.S**. contributed to the manuscript editing.

## Conflicts of Interest

The authors declare no conflicts of interest.

## Supporting information




**Supporting file**: adma74459‐sup‐0001‐SuppMat.docx.

## Data Availability

The data that support the findings of this study are available from the corresponding author upon reasonable request.

## References

[1] C. W. Li and M. W. Kanan , “CO_2_ Reduction at Low Overpotential on Cu Electrodes Resulting From the Reduction of Thick Cu_2_O Films,” Journal of the American Chemical Society 134 (2012): 7231–7234, 10.1021/ja3010978. 22506621

[2] C. O'Brien , R. K. Miao , S. Liu , et al., “Scal CO_2_ Regeneration,” ACS Energy Letters 6 (2021): 2952–5959.

[3] C.‐T. Dinh , T. Burdyny , and M. G. Kibria , “CO_2_ Electroreduction to Ethylene via Hydroxide‐Mediated Copper Catalysis at an Abrupt Interface,” Science 360 (2018): 783–787, 10.1126/science.aas9100. 29773749

[4] D. Salvatore and C. P. Berlinguette , “Voltage Matters When Reducing CO_2_ in an Electrochemical Flow Cell,” ACS Energy Letters 5 (2020): 215–220, 10.1021/acsenergylett.9b02356.

[5] M. Jouny , W. Luc , and F. Jiao , “Correction to “General Techno‐Economic Analysis of CO_2_ Electrolysis Systems”,” Industrial & Engineering Chemistry Research 59 (2020): 8121–8123, 10.1021/acs.iecr.0c01513.

[6] S. Park , I. Grigioni , and T. Alkayyali , “High Carbon Efficiency in CO‐to‐Alcohol Electroreduction Using a CO Reservoir,” Joule 7 (2023): 2335–2348, 10.1016/j.joule.2023.08.001.

[7] A. Ozden , J. Li , and S. Kandambeth , “Energy‐ and Carbon‐Efficient CO_2_/CO Electrolysis to Multicarbon Products via Asymmetric Ion Migration–adsorption,” Nature Energy 8 (2023): 179–190, 10.1038/s41560-022-01188-2.

[8] D. S. Ripatti , T. R. Veltman , and M. W. Kanan , “Carbon Monoxide Gas Diffusion Electrolysis That Produces Concentrated C_2_ Products With High Single‐Pass Conversion,” Joule 3 (2019): 2581–2581, 10.1016/j.joule.2019.09.012.

[9] R. Dorakhan , I. Grigioni , and B.‐H. Lee , “A Silver–copper Oxide Catalyst for Acetate Electrosynthesis From Carbon Monoxide,” Nature Synthesis 2 (2023): 448–457, 10.1038/s44160-023-00259-w.

[10] H. Yadegari , A. Ozden , and T. Alkayyali , “Glycerol Oxidation Pairs With Carbon Monoxide Reduction for Low‐Voltage Generation of C_2_ and C_3_ Product Streams,” ACS Energy Letters 6 (2021): 3538–3544, 10.1021/acsenergylett.1c01639.

[11] K. Xie , A. Ozden , R. K. Miao , Y. Li , D. Sinton , and E. H. Sargent , “Eliminating the Need for Anodic Gas Separation in CO_2_ Electroreduction Systems via Liquid‐to‐Liquid Anodic Upgrading,” Nature Communications 13 (2022): 3070, 10.1038/s41467-022-30677-x. PMC9163163 35654799

[12] E. Muller and S. Takegami , “Electrolytic Oxidation of Formaldehyde in Alkaline Solution,” Zeitschrift fuer Elektrochemie 34 (1928): 704–713.

[13] M. Yang , Y. Li , and Y. Zou et al., “Correlating the Valence state With the Adsorption Behavior of a Cu‐Based Electrocatalyst for Furfural Oxidation With Anodic Hydrogen Production Reaction,” Advanced Materials 35 (2023): 2304203, 10.1002/adma.202304203. 37354136

[14] H. Liu , N. Agrawal , and A. Ganguly , “Ultra‐Low Voltage Bipolar Hydrogen Production From Biomass‐Derived Aldehydes and Water in Membrane‐Less Electrolyzers,” Energy & Environmental Science 15 (2022): 4175–4189, 10.1039/D2EE01427K.

[15] Y. Pan , Y. Li , and C.‐L. Dong , “Unveiling the Synergistic Effect of Multi‐Valence Cu Species to Promote Formaldehyde Oxidation for Anodic Hydrogen Production,” Chemistry 9 (2023): 963–977, 10.1016/j.chempr.2022.12.008.

[16] F. Yang , S. Jiang , and S. Liu , “Dynamics of Bulk and Surface Oxide Evolution in Copper Foams for Electrochemical CO_2_ Reduction,” Communications Chemistry 7 (2024): 66, 10.1038/s42004-024-01151-0. PMC10978924 38548895

[17] S. Jiang , K. Klingan , C. Pasquini , and H. Dau , “New Aspects of Operando Raman Spectroscopy Applied to Electrochemical CO_2_ Reduction on Cu Foams,” Journal of Chemical Physics 150 (2019): 041718, 10.1063/1.5054109. 30709263

[18] Y. Deng , A. D. Handoko , and B. S. Yao , et al., “ *In Situ* Raman Spectroscopy of Copper and Copper Oxide Surfaces during Electrochemical Oxygen Evolution Reaction: Identification of Cu^III^ Oxides as Catalytically Active Species,” ACS Catalysis 4 (2016): 2473–2481.

[19] P. L. Cabot , R. C. Salvarezza , and A. J. Arvia , “The Influence of Slow Cu(OH)_2_ Phase Formation on the Electrochemical Behaviour of Copper in Alkaline Solutions,” Electrochimica Acta 33 (1988): 573–580.

[20] D. W. Shoesmith , T. E. Rummery , D. Owen , and W. Lee , “Anodic oxidation of copper in alkaline solutions 2—the open‐circuit potential behavior of electrochemically formed cupric hydroxide films,” Electrochimica Acta 22 (1977): 1403–1409.

[21] M. C. Biesinger , L. W. M. Lau , and A. R. Gerson , “Resolving Surface Chemical States in XPS Analysis of First Row Transition Metals, Oxides and Hydroxides: Sc, Ti, V, Cu and Zn,” Applied Surface Science 257 (2010): 887–898.

[22] D. Higgins , M. Wette , and B. M. Gibbons , “Copper Silver Thin Films With Metastable Miscibility for Oxygen Reduction Electrocatalysis in Alkaline Electrolytes,” ACS Applied Energy Materials 1 (2018): 1990–1999, 10.1021/acsaem.8b00090.

[23] K. Liu , M. Ma , and L. Wu , “Electronic Effects Determine the Selectivity of Planar Au–Cu Bimetallic Thin Films for Electrochemical CO_2_ Reduction,” ACS Applied Materials & Interfaces 11 (2019): 16546–16555, 10.1021/acsami.9b01553. PMC6509640 30969748

[24] D. Zhu , Y. Liu , M. Liu , X. Liu , P. N. Prasad , and M. T. Swihart , “Galvanic Replacement Synthesis of Multi‐Branched Gold Nanocrystals for Photothermal Cancer Therapy,” Journal of Materials Chemistry B 8 (2020): 5491–5499, 10.1039/D0TB00748J. 32478780

[25] P. Zhan , S. Yang , and M. Chu , “Amorphous Copper‐Modified Gold Interface Promotes Selective CO_2_ Electroreduction to CO,” ChemCatChem 14 (2022): 13, 10.1002/cctc.202200109.

[26] R. Daira , B. Boudjema , M. Bououdina , M. S. Aida , and C.‐D. Constantinescu , “Influence of Al Doping on the Physical Properties of CuO Thin Films,” Applied Sciences 13 (2023): 8193, 10.3390/app13148193.

[27] S. Xu , C. Wang , and D. Ren et al., “Tuning Intermediate Binding Enables Selective Electroreduction of Carbon Dioxide to Carbon Monoxide on a Copper–indium Catalyst,” Chemical Science 16 (2025): 8661–8672, 10.1039/d5sc01110h. PMC12062834 40353191

[28] M. Okada , Y. Tsuda , and K. Oka , “Experimental and Theoretical Studies on Oxidation of Cu‐Au Alloy Surfaces: Effect of Bulk Au Concentration,” Scientific Reports 6 (2016): 31101, 10.1038/srep31101. PMC4981884 27516137

[29] A. A. Mikhailova , A. P. Maltsev , P. C. D. Mendes , F. B. Zamudio , A. R. Oganov , and S. M. Kozlov , “Structure and Adsorption Properties of Cu–Au Nanoparticles in Harsh Reactive Environments,” Journal of Chemical Physics 163 (2025): 124302, 10.1063/5.0288099. 40981451

[30] H. Mahajan and S. Cho , “Novel Au Nanorod/Cu2O Composite Nanoparticles for a High‐Performance Supercapacitor,” RSC Advances 12 (2022): 9112–9120. 10.1039/d2ra00812b PMC8985136 35424862

[31] Y. Zha , M. Liu , J. Wang , et al., “Electrochemical Ammonia Synthesis by Reduction of Nitrate on Au Doped Cu Nanowires,” RSC Advances 13 (2023): 9839–9844. 10.1039/d3ra00679d PMC10043758 36998524

[32] Y. Zha , Y. Zhang , X. Yang , et al., “Tuning Intermediates' adsorption for Efficient Electrosynthesis of Urea From Carbon Dioxide and Nitrate via Doping Au into Cu,” Journal of Materials Chemistry A 13 (2025): 16596–16605.

[33] S. Nakayama , T. Kaji , T. Notoya , and T. Osakai , “Mechanistic Study of the Reduction of Copper Oxides in Alkaline Solutions by Electrochemical Impedance Spectroscopy,” Electrochimica Acta 53 (2008): 3493–3499, 10.1016/j.electacta.2007.12.019.

[34] V. Maurice , H.‐H. Strehblow , and P. Marcus , “In Situ STM Study of the Initial Stages of Oxidation of Cu(111) in Aqueous Solution,” Surface Science 458 (2000): 185–194.

[35] Z. Zhang , X. Huang , Z. Chen , et al., “Membrane Electrode Assembly for Electrocatalytic CO_2_ Reduction: Principle and Application,” Angewandte Chemie 62 (2023): 202302789. 10.1002/anie.202302789 36971005

[36] Y. Ren , Z. Ma , L. Qian , S. Dai , H. He , and P. G. Bruce , “Ordered Crystalline Mesoporous Oxides as Catalysts for CO Oxidation,” Catalysis Letters 131 (2009): 146–154, 10.1007/s10562-009-9931-0.

[37] I. Fouzaï , S. Gentil , V. C. Bassetto , et al., “Catalytic Layer‐Membrane Electrode Assembly Methods for Optimum Triple Phase Boundaries and Fuel Cell Performances,” Journal of Materials Chemistry A 9 (2021): 11096–11123.

[38] T. Soboleva , K. Malek , Z. Xie , T. Navessin , and S. Holdcroft , “PEMFC Catalyst Layers: The Role of Micropores and Mesopores on Water Sorption and Fuel Cell Activity,” ACS Applied Materials & Interfaces 3 (2011): 1827–1837, 10.1021/am200590w. 21574609

[39] W. Sugimoto , H. Iwata , K. Yokoshima , Y. Murakami , and Y. Takasu , “Proton and Electron Conductivity in Hydrous Ruthenium Oxides Evaluated by Electrochemical Impedance Spectroscopy: the Origin of Large Capacitance,” Journal of Physical Chemistry B 109 (2005): 7330–7338, 10.1021/jp044252o. 16851839

[40] M. Itagaki , S. Suzuki , I. Shitanda , K. Watanabe , and H. Nakazawa , “Impedance Analysis on Electric Double Layer Capacitor With Transmission Line Model,” Journal of Power Sources 164 (2007): 415–424, 10.1016/j.jpowsour.2006.09.077.

[41] Z. Xing , L. Hu , D. S. Ripatti , X. Hu , and X. Feng , “Enhancing Carbon Dioxide Gas‐Diffusion Electrolysis by Creating a Hydrophobic Catalyst Microenvironment,” Nature Communications 12 (2021): 136, 10.1038/s41467-020-20397-5. PMC7794506 33420043

[42] S. Subramanian , J. Kok , and P. Gholkar , “CO Residence Time Modulates Multi‐Carbon Formation Rates in a Zero‐Gap Cu Based CO_2_ Electrolyzer,” Energy & Environmental Science 17 (2024): 6728–6738, 10.1039/D4EE02004A.

[43] R. Chen , H. Y. Su , D. Liu , et al., “Highly Selective Production of Ethylene by Electroreduction of Carbon Monoxide,” Angewandte Chemie 59 (2019): 1. 10.1002/anie.201910662 31697030

[44] J. Li , Z. Wang , and C. McCallum , “Constraining CO Coverage on Copper Promotes High‐Efficiency Ethylene Electroproduction,” Nature Catalysis 2 (2019): 1124–1131, 10.1038/s41929-019-0380-x.

[45] X. Chang , J. Li , and H. Xiong , “C−C Coupling Is Unlikely to Be the Rate‐Determining Step in the Formation of C^2+^ Products in the Copper‐Catalyzed Electrochemical Reduction of CO,” Angewandte Chemie International Edition 61 (2022): 2, 10.1002/anie.202111167. 34779566

[46] D. Verboekend and J. Pérez‐Ramírez , “Desilication Mechanism Revisited: Highly Mesoporous all‐Silica Zeolites Enabled through Pore‐Directing Agents,” Chemistry‐A European Journal 17 (2011): 1137–1147, 10.1002/chem.201002589. 21243680

[47] M. R. Singh , Y. Kwon , Y. Lum , J. W. Ager , and A. T. Bell , “Hydrolysis of Electrolyte Cations Enhances the Electrochemical Reduction of CO_2_ over Ag and Cu,” Journal of the American Chemical Society 138 (2016): 13006–13012, 10.1021/jacs.6b07612. 27626299

[48] G. Kresse and J. Hafner , “Ab Initio Molecular Dynamics for Liquid Metals,” Physical Review B 47 (1993): 558–561, 10.1103/PhysRevB.47.558. 10004490

[49] J. P. Perdew , K. Burke , and M. Ernzerhof , “Generalized Gradient Approximation Made Simple,” Physical Review Letters 77 (1996): 3865–3868, 10.1103/PhysRevLett.77.3865. 10062328

[50] S. Grimme , S. Ehrlich , and L. Goerigk , “Effect of the Damping Function in Dispersion Corrected Density Functional Theory,” Journal of Computational Chemistry 32 (2011): 1456–1465, 10.1002/jcc.21759. 21370243

[51] S. Grimme , J. Antony , S. Ehrlich , and H. Krieg , “A Consistent and Accurate Ab Initio Parametrization of Density Functional Dispersion Correction (DFT‐D) for the 94 Elements H‐Pu,” Journal of Chemical Physics 132 (2010): 154104, 10.1063/1.3382344. 20423165

[52] S. M. R. Islam , F. Khezeli , S. Ringe , and C. Plaisance , “An Implicit Electrolyte Model for Plane Wave Density Functional Theory Exhibiting Nonlinear Response and a Nonlocal Cavity Definition,” Journal of Chemical Physics 159 (2023): 234117. 10.1063/5.0176308 38112507

[53] G. Kresse and J. Furthmüller , “Efficient Iterative Schemes for Ab Initio Total‐Energy Calculations Using a Plane‐Wave Basis Set,” Physical Review B 54 (1996): 11169–11186, 10.1103/PhysRevB.54.11169. 9984901

