# Supplementary material for: Gold‐Stabilized Copper Enables Anodic Hydrogen Evolution for Ultralow‐Voltage CO‐to‐Ethylene Electrolysis
Source: Adv Mater. 2026 Aug 9;38(56):e74459. doi: 10.1002/adma.74459 (PMC13648890; doi:10.1002/adma.74459)
Supplement: Supplementary file 1 [file ADMA-38-e74459-s001.docx]

**Supplementary Information**

**Figure S1. Comparative analysis of electrochemical performance and product formation using Cu foam and OD-Cu catalyst.** (a) Linear sweep voltammetry (LSV) curve for copper foam and oxide-derived copper (OD-Cu). (b) Molar quantity of liquid products from anolyte and (c) Faradaic efficiency for the anode product when using copper foam and OD-Cu, conducted at 0.45 V vs. RHE in a solution of 1 M KOH with an addition of 250 mM furfural. The A-HER was run for 800 s with Cu foam and 1500 s with OD-Cu. Note that the transformation of furfural to furoic acid occurs through a non-Faradaic reaction, specifically the Cannizzaro reaction, in an alkaline medium. This is the underlying reason for the increased production of furoic acid-derived products compared to the moles of hydrogen evolved in **Figure S1b**.

**Figure S2.** (a) Linear sweep voltammetry (LSV) curves depicting the electrochemical behavior of OD-Cu, PtCu, and AuCu catalysts. (b) Quantification of the molarity of liquid products in the anolyte, obtained from OD-Cu, AuCu, and PtCu catalysts under potential of 0.4 V_RHE_ for 1200 s. These measurements were conducted in a solution containing 1 M KOH with the addition of 250 mM furfural. Additionally, the molarities of furoic acid and furfuryl alcohol resulting from the Cannizzaro reaction, a non-Faradaic process, are also provided. (c) Quantification of the molarity of liquid products in the anolyte, obtained from OD-Cu, AuCu, and PtCu catalysts under potential of 0.4 V_RHE_ for 1200 s. These measurements were conducted in a solution containing 1 M KOH with the addition of 200 mM formaldehyde. Additionally, the molarities of formic acid and methanol resulting from the Cannizzaro reaction, a non-Faradaic process, are also provided. (d) Faradaic efficiency for the anode product when using AuCu under different current density in a solution of 1 M KOH with an addition of 200 mM formic acid.

**Figure S3.** Cyclic voltammetry (CV) profiles illustrating the electrochemical behavior of (a) Cu foam, (b) OD-Cu, (c) OD-Cu after electrolysis, (d) PtCu, (e) AuCu, and (f) AuCu after electrolysis, all conducted in a solution of 1 M KOH. The "post electrolysis" samples refer to the catalysts utilized for the anodic hydrogen evolution reaction (a-HER) prior to CV analysis.

**Figure S4.** Powder X-ray diffraction (XRD) patterns of (a) AuCu and (b) OD-Cu at the initial stage and under open-circuit potential (OCP) conditions. Identified diffraction peaks include Cu_2_O at 2θ (hkl) – 29.7° (011), 36.6° (111), 42.5° (002), 52.7° (112), 61.7° (022), 73.9° (113); Cu at 43.3° (111), 50.5° (002), 74.2° (022); CuO at 36.6° (111), 42.6° (002), 61.8° (022), 74.0° (113); CuCl at 28.5° (111), 47.4° (022), 56.3° (113), 76.6° (133); Au at 38.3° (111), 44.5° (002), 64.7° (022); and Au₀.₈Cu₀.₂ at 38.8° (111), 45.2° (002), 65.7° (022).^2-6^ The presence of CuCl results from the galvanic exchange between the gold precursor and copper; however, it is completely washed away under operando conditions.

**Figure S5.** Scanning electron microscopy (SEM) images of the anode: Top view of (a) AuCu, (b) PtCu and (c) OD-Cu and top view of post-electrolysis of (d) AuCu and (e) OD-Cu.

**Figure S6.** Energy-dispersive X-ray (EDX) analysis mapping of the post-electrolysis catalyst from SEM: (a) AuCu (b) PtCu and (c) OD-Cu.

**Figure S7.** Energy-dispersive X-ray (EDX) spectra and atomic percentages associated with the EDX mapping in **Figure S6** for (a) OD-Cu and (b) AuCu post-electrolysis catalysts. *Net Int.* represents the background-subtracted X-ray signal intensity. *Error %* denotes the relative measurement uncertainty. *Z* accounts for atomic number corrections, *A* compensates for X-ray absorption effects, and *F* corrects for secondary fluorescence interference.

**Figure S8.** Transmission electron microscopy (TEM) images of OD-Cu and AuCu post-electrolysis catalysts. (a) Bright-field and (b) dark-field images of OD-Cu. EDX mapping showing (c) oxygen (O-K) and (d) copper (Cu-K) distributions in OD-Cu. (e) Bright-field and (f) dark-field images of AuCu. EDX elemental maps showing (g) gold (Au-L) and (h) copper (Cu-K) distributions in the AuCu catalyst.

**Figure S9.** (a) Linear sweep voltammetry (LSV) curves for the cathodic COR and anodic HER (a-HER) system with varying Au loading on AuCu catalyst anodes, using CuO and C-support_3. For example, Au₁Cu₁₀ represents a 1:10 atomic ratio of Au to Cu. (b) Comparison of anodic product distribution across different Au loadings at 200 mA/cm^2^ current density.

**Figure S10.** Characterization of various commercial C-support with different sizes and synthesis temperatures. Detailed information regarding characterization and performance metrics of the carbon supports can be found in **Table S3**. (a) Pore-size distribution calculated by Barrett-Joyner-Halenda (BJH) method from desorption branch of isotherm. (b) Electrochemical impedance spectroscopy (EIS) results obtained for carbon support. (c) Plot depicting the relationship between double-layer capacitance and Brunauer-Emmett-Teller (BET) surface area.

**Figure S11.** High-resolution transmission electron microscopy (HR-TEM) and EDX mapping of the post-electrolysis cathode, comprising copper (II) oxide, and the following carbon supports: (a) C-support_1, (b) C-support_2, (c) C-support_3, (d) C-support_4, and (e) C-support_5.

**Figure S12.** Comparative analysis of product distribution under varying current densities and single-pass conversion efficinecy (SPCE) for different CuO catalysts and C-support_3, conducted under limited CO flow conditions. Detailed information regarding flow rates can be found in **Table S1**.

**Figure S13.** Comparative analysis of product distribution under CO flow for different carbon support & CuO catalysts: (a) CuO & C-support_1, (b) CuO & C-support_2, (c) CuO & C-support_3, (d) CuO & C-support_4, and (e) CuO & C-support_5. Detailed information on carbon supports is provided in **Table S3**. The COR was performed in 1M KOH at current density of 200 mA/cm^2^.

**Figure S14.** Comparative analysis of product distribution for copper catalyst with different oxidation states. The COR was performed in 1M KOH at current density of 200 mA/cm^2^.

**Figure S15.** Comparative analysis of product distribution for CuO and C-support_3 with different weight ratio. The COR was performed in 1M KOH at current density of 200 mA/cm^2^.

**Figure S16.** Comparative analysis of ethylene selectivity under different partial pressure of CO in syngas (H_2_ + CO) normal flow: (a) CuO & C-support_1, (b) CuO & C-support_2, (c) CuO & C-support_3, (d) CuO & C-support_4, and (e) CuO & C-support_5. Detailed information on carbon supports is provided in **Table S3**. The COR was performed in 1M KOH at current density of 200 mA/cm^2^.

**Table S1.** Integrated performance benchmarking of CO/CO₂-to-C₂ electrolysis systems using OER-coupled and alternative anodic reactions.

| **Catalyst** | **Reactant** | **Anode reaction** | **Cell voltage** | **Current density** | **Product FE** | **SPCE / carbon efficiency** | **Stability** | **Ref.** |
| --- | --- | --- | --- | --- | --- | --- | --- | --- |
| Ag-Ru-Cu | CO | OER | 2.65 V | 300 mA cm⁻² | 93% C₂+ | 85% | 100 h | 14 |
| CuO | CO | OER | 2.18 V | 200 mA cm⁻² | 90% C₂+ | N/A | 110 h | 57 |
| Cu-Ag/DA | CO | OER | 2.60 V | 100 mA cm⁻² | 90% C₂+ | 71% | 800 h | 58 |
| Ag-Cu₂O | CO | OER | 2.50 V | 100 mA cm⁻² | 90% C₂+ | N/A | 18 h | 59 |
| Cu | CO | OER | 2.32 V | 140 mA cm⁻² | 71% C₂+ | 43% | 24 h | 9 |
| Cu:Py:SSC | CO | OER | 2.41 V | 120 mA cm⁻² | 76% C₂+ | 27% | 110 h | 7 |
| Cu-CCBH | CO | OER | 2.23 V | 240 mA cm⁻² | 87% C₂+ | 95% | 200 h | 8 |
| OD-Cu | CO | OER | 3.25 V | 500 mA cm⁻² | 90% C₂+ | N/A | 1 h | 60 |
| Cu-NN | CO₂/CO | OER | 2.30 V | 180 mA cm⁻² | 86% C₂+ | ~50% | 100 h | 11 |
| Cu, porous separator | CO | OER | 1.95 V | 200 mA cm⁻² | 96% C₂+ | 53% | 250 h | 61 |
| Cu, porous separator | CO | OER | 1.95 V | 200 mA cm⁻² | 91% C_2+_ | 97% | N/A | 61 |
| Cu/Pt-CC | CO | Glycerol oxidation | 1.34 V | 180 mA cm⁻² | 51% C2H4 / 71% C_2+_ | N/A | 50 h | 10 |
| Cu/Pt | CO₂ | Glucose oxidation | 1.90 V | 100 mA cm⁻² | 42% C_2+_ / 32% C_2_H_4_ | ~45-48% carbon efficiency | >80 h | 62 |
| **CuO/Carbon + AuCu** | **CO** | **Furfural-assisted a-HER** | **0.73 V** | **200 mA cm⁻²** | **55% C_2_H_4_** | **91%** | **-** | **This work** |
| **CuO/Carbon + AuCu** | **CO** | **Furfural-assisted a-HER** | **0.92 V** | **400 mA cm⁻²** | **63% C_2_H_4_ / ~87% C_2+_** | **93%** | **75 h** | **This work** |

Note: The two rows for this work correspond to two representative COR-a-HER operating points. The dash in the 200 mA cm⁻² row indicates that an extended stability test was not assigned to that operating point; the 75 h entry corresponds to the 400 mA cm⁻² stability data.

**Table S2**. Flow rate of CO in the low-flow CO reduction experiment for CuO & C-support_3 catalyst.

|  | Current density (mA/cm^2^) | 100 | 200 | 300 | 400 |
| --- | --- | --- | --- | --- | --- |
| CO flow (sccm) | Normal flow | 20 | 20 | 20 | 20 |
|  | Slow flow | 0.42 | 0.82 | 1.15 | 1.54 |

**Figure S17.** (a) Linear sweep voltammetry (LSV) curves depicting the anodic hydrogen evolution reaction (a-HER) system, utilizing AuCu catalyst anodes with CuO and C-support_3, while employing different cations. (b) Comparative analysis of product distribution under various current densities, cations, and cell voltages and 20 sccm of CO flow.

**Table S3**. Table displaying cell voltage, ethylene faradaic efficiency (FE), and corresponding electricity energy demand. The cell voltage and ethylene FE were obtained at 200 mA/cm^2^ from the normal flow (20 sccm) CO reduction system with using AuCu as anode.

|  | Cell voltage (V) | Ethylene FE (%) | Electricity energy demand (GJ / tonne ethylene) |
| --- | --- | --- | --- |
| 1M KOH | 0.82 | 42 | 53.7 |
| 1M NaOH | 1.14 | 50 | 62.7 |
| 1M LiOH | 1.29 | 56 | 63.4 |

**Table S4**. Faradaic efficiency (FE) of hydrogen, acetate, ethanol, propanol and furoic acid during the stability test using CuO & C-support_3 under a-HER conditions with low CO flow.

|  | | | FE of cathode product | | | | | FE of anode product | |
| --- | --- | --- | --- | --- | --- | --- | --- | --- | --- |
| Count | Time (s) | Current density (mA/cm^2^) | Hydrogen FE (%) | Ethylene FE  (%) | Acetate FE (%) | Ethanol FE (%) | Propanol FE (%) | Hydrogen FE (%) | Furoic acid FE (%) |
| 1 | 3450 | 223 | 9 | 58 | 12 | 13 | 5 | 98 | 97 |
| 2 | 7332 | 235 | 13 | 54 | 9 | 12 | 7 | 103 | 99 |
| 3 | 13545 | 306 | 8 | 59 | 11 | 14 | 7 | 96 | 101 |
| 4 | 16170 | 250 | 12 | 56 | 10 | 7 | 8 | 89 | 87 |
| 5 | 22769 | 251 | 11 | 57 | 13 | 8 | 4 | 92 | 94 |
| 6 | 28052 | 260 | 15 | 59 | 15 | 6 | 3 | 99 | 93 |
| 7 | 30580 | 268 | 14 | 57 | 14 | 15 | 6 | 94 | 96 |

**Table S5**. Physical characteristic of C-supports used in cathode.

| Sample number | Commercial name | Particle size (nm) | Surface area (m^2^/g) | Morphology | Type |
| --- | --- | --- | --- | --- | --- |
| C-support_1 | Acetylene black | 123 | 80 | Sphere | Carbon black |
| C-support_2 | Vulcan XC-72R | 50 | 244 | - | Carbon black |
| C-support_3 | Bamboo | 30 | 725 | Sphere | Activated carbon |
| C-support_4 | Super P conductive | 40 | 66 | Sphere | Carbon black |
| C-support_5 | D10 | 30 | 554 | Graphite |  |

**Table S6**. Crossover of COR liquid products after 30 minutes of electrolysis at 200 mA/cm² using AEM and CEM. Sustainion X37-50 RT was used as the AEM, and Nafion XL as the CEM. The electrolyte was 1M KOH.

|  | Cathode | | | Anode | | |
| --- | --- | --- | --- | --- | --- | --- |
| FE (%) | Acetate | Ethanol | Propanol | Acetate | Ethanol | Propanol |
| AEM | 6 | 5 | 2 | 5 | 14 | 8 |
| CEM | 8 | 10 | 6 | 2 | 8 | 3 |

1. **Supplementary Note 1**

**Voltage breakdown analysis**

**Figure S18.** Schematic cell figure of voltage-breakdown analysis system.

**Figure S19.** Nyquist plot of the (a) C-support_3 & CuO for the cathode and (b) AuCu foam for the anode impedance for different current density.

We used an analytical cell incorporating reference electrodes on both sides of the membrane, enabling the direct measurement of cathodic and anodic overpotentials in zero- and small-gap electrolyzers (**Figure S18**).^33-35^ For the membrane electrode assembly (MEA), we employed a commercial Sustainion X 37-50 RT anion exchange membrane (AEM). The cathode consisted of a CuO & C-support_3 catalyst, while the oxygen evolution reaction (OER) was facilitated by iridium oxide on TiO₂ felt. The anode catalyst for the aldehyde hydrogen evolution reaction (a-HER) was AuCu foam. Electrochemical measurements were conducted using an Autolab electrochemical workstation (PGSTAT204 equipped with a 10A booster) in both MEA and slim flow cell configurations. All experiments were repeated three times to report the average and standard error. Electrolysis was performed for at least 30 minutes before conducting electrochemical impedance spectroscopy (EIS).

Throughout the experiments, CO2 or CO was supplied to the electrolyzer at a controlled flow rate of 20 sccm using a mass flow controller. The anode side was supplied with different electrolytes depending on the specific reaction conditions: 0.01 M H_2_SO_4_ for acidic CO_2_ reduction (CO_2_R), 0.1 M KHCO_3_ for neutral CO_2_R, and 1 M KOH for carbon monoxide reduction (COR).

The ohmic drop within the cell and between the working and reference electrodes was determined using potentiostatic EIS against an Ag/AgCl reference electrode (**Figure S19**). The resistance value was obtained from the intersection of the curve with the real impedance (Re, Ω) axis in the Nyquist plot. iR correction was applied post-electrolysis for all measurements. All reported potentials were converted to the reversible hydrogen electrode (RHE) scale using the following equation:

$$E_{RHE}=E_{\left( \frac{Ag}{Agcl} \right)}+0.21 V+0.0592 V\times local pH$$

And we can estimate E_others_ from following equation

$$E_{cell}=E_{anode}+E_{thermodynamic}+E_{cathode}++E_{Nernstian pH loss}+E_{others}$$

$$E_{anode}=V_{anode vs. RHE}-V_{anode thermodynamic potential}$$

$$V_{anode vs. RHE}=\left( V_{anode vs.\frac{Ag}{Agcl}}-R_{Ohmic}*I \right)+E_{Ag/Agcl}^{0}+0.059*({pH}_{anode surface})$$

$$E_{cathode}=V_{cathode vs RHE}-V_{cathode thermodynamic potential}$$

$$V_{cathode vs. RHE}=\left( V_{cathode vs.\frac{Ag}{Agcl}}-R_{Ohmic}*I \right)+E_{Ag/Agcl}^{0}+0.059*({pH}_{cathode surface})$$

$$E_{Nernstian pH loss}=0.059\times({pH}_{cathode surface}-{pH}_{anode surface})$$

Given the experimental challenges associated with directly measuring local pH near the electrode surface in enclosed configurations such as membrane electrode assemblies (MEAs), the local pH values at both the cathode and anode were obtained through COMSOL simulations. It should also be noted that in **Figure 1b**, E_others_ includes both overpotential losses and pH-dependent Nernstian shifts, aiming a clearer comparison of cell voltage across different electrolyzer systems. The parameters described above are summarized in **Table S7**.

**Table S7.** Breakdown of cell voltage in neutral/acidic CO_2_R and COR systems.

| System | E_cell_ | E_cathode_ | E_anode_ | E_thermodynamic_ | E_Nernstian pH loss_ | pH_cathode_ | pH_anode_ |
| --- | --- | --- | --- | --- | --- | --- | --- |
| Neutral CO_2_R | 3.7 | 0.89 | 0.74 | 1.23 | 0.56 | 12.5 | 3 |
| Acidic CO_2_R | 3.8 | 1.4 | 0.47 | 1.23 | 0.56 | 13 | 3 |
| COR – OER | 2.3 | 0.55 | 0.46 | 1.23 | 0.03 | 14 | 13.8 |
| COR – aHER | 0.73 | 0.55 | 0.007 | 0.05 | 0.03 | 14 | 13.8 |

2. **Supplementary Note 2**

**Structural and chemical understanding of the AuCu anode**

The AuCu anode was examined using complementary structural, chemical-state, and electrochemical measurements to establish how Au is incorporated into the oxide-derived Cu surface and how this modification affects the Cu redox behavior under anodic hydrogen evolution reaction (a-HER) conditions. The combined GI-XRD, SEM-EDS, TEM-EDS, Au 4f XPS, Au L3-edge XAS, Cu 2p XPS, Cu LMM Auger, CV, and LSV analyses indicate that the AuCu catalyst is best described as Au-modified OD-Cu containing Au-rich surface domains together with local Au-Cu interfacial motifs. These Au-containing domains remain detectable after electrolysis and are associated with a more stable low-valent Cu surface under anodic conditions.

**Au incorporation chemistry**

The AuCu anode was prepared by exposing OD-Cu foam to HAuCl_4_ solution without a subsequent high-temperature alloying step. Under these conditions, Au deposition is consistent with surface-limited galvanic replacement between AuCl_4_^−^ and surface Cu/Cu^+^ species. In this process, AuCl_4_^−^ is reduced to metallic Au, while Cu is oxidized and may dissolve or form transient chloride-containing species. This chemistry is consistent with established galvanic replacement processes involving noble-metal deposition on Cu-based surfaces.^47,48^ A simplified reaction for metallic Cu can be written as:

$$2Au{Cl}^{4-}+3Cu\left( s \right) \to2Au\left( s \right)+3\mathrm{Cu}^{2+}+8{Cl}^{-}$$

Because the Au treatment is conducted at low temperature and without thermal alloying, the resulting structure is expected to be dominated by surface-deposited Au-containing domains and local Au-Cu interfacial regions rather than homogeneous bulk alloying throughout the Cu foam. Grazing-incidence X-ray diffraction (GI-XRD) was performed at an incident angle of 0.6^0^ to enhance the surface sensitivity of the diffraction measurement. Compared with conventional X-ray diffraction, this geometry reduces the X-ray penetration depth and preferentially probes crystalline phases in the near-surface region of the Cu foam. The GI-XRD patterns therefore provide surface-sensitive information on crystalline Cu, Cu oxide, and Au-containing phases (**Figure S20**). However, because diffraction requires sufficient long-range crystalline order, this measurement does not resolve atomically dispersed Au species, very small Au clusters, or non-crystalline local Au–Cu interfacial motifs. The Au-containing diffraction features are therefore assigned to near-surface metallic Au and/or Au–Cu alloy-like crystalline domains, while the local Au coordination environment is further evaluated using XPS, XAS, and electron microscopy.^2-6,47,48^

**Figure S20**. GI-XRD patterns of AuCu before and after electrolysis. GI-XRD patterns of AuCu in the pre-reaction and post-reaction states. The diffraction peaks are assigned to Cu, Cu_2_O, Au, and minor CuO-related phases based on reported Cu, Cu oxide, Au, and Au-Cu diffraction references^2-6^. Au-containing diffraction features remain after a-HER operation, indicating the retention of Au-containing crystalline domains on the catalyst. The GI-XRD results support the coexistence of Au-containing domains with Cu-derived phases, while the detailed local Au coordination environment is further evaluated using XPS, XAS, and electron microscopy.

**Figure S21**. SEM–EDS elemental mapping of AuCu before and after electrolysis.

(a–d) Pre-electrolysis and (e–h) post-electrolysis SEM image and corresponding EDS elemental maps of AuCu. Panels (a) and (e) show the SEM images, (b) and (f) show Au maps, (c) and (g) show C maps, and (d) and (h) show O maps. The results confirm that Au remains distributed on the catalyst surface after electrolysis, while the overall morphology of the AuCu catalyst is largely preserved.

**Figure S22**. SEM images of OD-Cu before and after a-HER electrolysis.

(a,b) SEM images of OD-Cu before electrolysis and (c,d) after a-HER electrolysis. The OD-Cu catalyst shows a nanowire-like oxide-derived morphology before reaction, while noticeable surface reconstruction and morphological roughening are observed after a-HER operation.

**Au chemical state and retention after operation**

High-resolution Au 4f XPS was used to evaluate the surface Au species before and after electrolysis. The Au 4f doublet remains detectable after a-HER operation, confirming that Au species are retained on the catalyst surface. The weak Au 4f signal is consistent with the low surface Au content of the Au-modified Cu foam. The detectable Au 4f features are assigned mainly to metallic Au/Au-Cu surface species, consistent with the expected product of galvanic replacement between AuCl_4_^−^ and surface Cu/Cu^+^ species.^30,32,47,48^

Au L3-edge XAS further indicates that Au is present predominantly in a reduced metallic Au-like state. The XANES spectra of AuCu are closer to Au foil than to Au(III) oxide references, and the Au-related EXAFS features are retained after a-HER operation. These results show that Au remains in the catalyst after electrolysis and does not convert primarily into oxidized Au species. Because ensemble-averaged XAS can include contributions from Au-rich domains, Au clusters, and local Au-Cu coordination environments, the XAS data are used here to establish Au retention and oxidation state rather than to assign a single unique Au coordination structure.^30,32,47,48^

**Figure S23**. Au 4f XPS spectra of AuCu before and after electrolysis. Au 4f spectra of AuCu in the as-synthesized and post-electrolysis states. The characteristic Au 4f doublet remains detectable after operation, confirming that surface Au species are retained during a-HER. The weak signal intensity reflects the low surface Au content on the Au-modified Cu foam.

**Figure S24**. Au L3-edge XAS analysis of AuCu catalysts. (a) Au L₃-edge XANES spectra and (b) Fourier-transformed EXAFS spectra of AuCu in the as-synthesized, OCP-treated, and post-a-HER states, compared with Au foil and Au (III) oxide references. The AuCu spectra are closer to metallic Au than Au (III) oxide, indicating that Au is mainly present in a reduced metallic state. The retained Au signal after a-HER confirms the persistence of Au-containing surface domains during electrolysis.

**Cu oxidation-state stabilization by Au modification**

The effect of Au modification on the Cu redox state was evaluated using high-resolution Cu 2p XPS together with Cu LMM Auger analysis. Cu 2p spectra provide information on Cu^2+^-associated shake-up features and oxide/hydroxide formation, but Cu^0^ and Cu^+^ are difficult to distinguish using Cu 2p alone. Therefore, Cu LMM spectra were used as a complementary chemical-state constraint to distinguish Cu0, Cu_2_O/Cu^+^, CuO/Cu^2+^, and Cu(OH)_2_/Cu^2+^ contributions, following established Cu XPS/Auger analysis procedures.^1^

The Cu 2p and Cu LMM fitting results show that OD-Cu undergoes substantial oxidation/hydroxylation after anodic conditioning, especially at +0.5 V vs. RHE. In contrast, AuCu retains a larger low-valent Cu fraction after the same treatment. In the Cu 2p analysis, OD-Cu at +0.5 V contains a dominant Cu2+ contribution, whereas AuCu maintains a substantial low-valent Cu component. The Cu LMM analysis gives the same qualitative trend: OD-Cu after +0.5 V is dominated by Cu(OH)_2_/CuO-like species, while AuCu retains a larger Cu_2_O/Cu^+^-rich contribution. These results indicate that Au modification suppresses deep surface hydroxylation/oxidation of Cu and stabilizes low-valent Cu species under a-HER-relevant anodic potentials. This trend is consistent with the operando Raman and in-situ Cu XAS results, which show stronger potential-dependent Cu-O(H) evolution on OD-Cu and a more stable low-valent Cu-O motif on AuCu.^23-30,56^

**Figure S25**. Cu 2p XPS fitting of OD-Cu and AuCu catalysts.

Deconvoluted Cu 2p spectra of OD-Cu and AuCu catalysts measured under different pretreatment/conditioning conditions: (a) OD-Cu as-synthesized, (b) AuCu as-synthesized, (c) OD-Cu after OCP treatment, (d) AuCu after OCP treatment, (e) OD-Cu after electrochemical conditioning at +0.3 V vs. RHE, (f) AuCu after electrochemical conditioning at +0.3 V vs. RHE, (g) OD-Cu after electrochemical conditioning at +0.5 V vs. RHE, and (h) AuCu after electrochemical conditioning at +0.5 V vs. RHE. The fitted components are assigned to low-valent Cu^0^/Cu^+^, CuO/Cu^2+^, and Cu(OH)_2_/Cu^2+^ species, with shake-up satellite features included for Cu^2+^. Because Cu^0^ and Cu^+^ overlap strongly in the Cu 2p region, the Cu 2p fitting is interpreted together with the Cu LMM Auger analysis in **Figure S26** and **Table S9**. OD-Cu shows an increased Cu^2+^/Cu(OH)_2_ contribution after anodic conditioning, especially at +0.5 V vs. RHE, whereas AuCu retains a larger low-valent Cu fraction under the same conditions, supporting Au-assisted stabilization of the Cu surface under a-HER-relevant anodic potentials.

**Table S8**. Cu 2p fitting results for OD-Cu and AuCu. Values represent relative fitted contributions from the deconvoluted Cu 2p spectra. Because Cu^0^ and Cu^+^ overlap in the Cu 2p region, the assignments are interpreted together with the Cu LMM analysis in **Table S9**.

| **Sample** | **Cu^0^ (%)** | **Cu_2_O/Cu^+^ (%)** | **CuO/Cu^2+^ (%)** | **Cu(OH)_2_/Cu^2+^ (%)** | **Low-valent Cu (%)** | **Cu^2+^ total (%)** |
| --- | --- | --- | --- | --- | --- | --- |
| OD-Cu as synthesized | 15.0 | 85.0 | 0.0 | 0.0 | 100.0 | 0.0 |
| AuCu as synthesized | 30.2 | 18.6 | 12.1 | 39.0 | 48.9 | 51.1 |
| OD-Cu OCP | 15.0 | 85.0 | 0.0 | 0.0 | 100.0 | 0.0 |
| OD-Cu +0.3 V | 14.3 | 80.9 | 0.0 | 4.8 | 95.2 | 4.8 |
| OD-Cu +0.5 V | 2.4 | 9.1 | 6.3 | 82.2 | 11.5 | 88.5 |
| AuCu OCP | 52.6 | 42.4 | 1.9 | 3.1 | 95.0 | 5.0 |
| AuCu +0.3 V | 74.7 | 13.2 | 3.7 | 8.4 | 87.8 | 12.2 |
| AuCu +0.5 V | 63.8 | 14.2 | 3.9 | 18.0 | 78.1 | 21.9 |

**Figure S26**. Cu LMM Auger fitting of OD-Cu and AuCu catalysts.

Deconvoluted Cu LMM spectra of (a,c,e) OD-Cu and (b,d,f) AuCu in the (a,b) as-synthesized state, (c,d) after OCP treatment, and (e,f) after +0.5 V vs. RHE conditioning. The fitted components are assigned to Cu^0^, Cu_2_O/Cu+, CuO/Cu²⁺, and Cu(OH)_2_/Cu^2+^ species. Compared with OD-Cu, which shows a larger Cu^2+^ oxide/hydroxide contribution after anodic conditioning, AuCu retains a higher Cu_2_O/Cu^+^-rich low-valent Cu fraction, supporting Au-assisted stabilization of the Cu surface under a-HER-relevant anodic potentials.

**Table S9**. Cu LMM fitting results for OD-Cu and AuCu. Cu LMM spectra were used as a complementary chemical-state constraint because Cu_0_ and Cu_+_ are not reliably separated using Cu 2p alone.

| **Sample** | **R^2^** | **Cu(OH)_2_ (%)** | **Cu_2_O (%)** | **CuO (%)** | **Cu^0^ (%)** | **Cu^2+^ total (%)** |
| --- | --- | --- | --- | --- | --- | --- |
| AuCu as-synthesized | 0.981 | 22.4 | 0.0 | 38.8 | 38.8 | 61.2 |
| AuCu OCP | 0.991 | 0.0 | 92.3 | 7.7 | 0.0 | 7.7 |
| AuCu +0.5 V | 0.981 | 18.9 | 64.2 | 16.8 | 0.0 | 35.8 |
| OD-Cu as-synthesized | 0.977 | 7.9 | 0.0 | 85.2 | 7.0 | 93.0 |
| OD-Cu OCP | 0.985 | 16.6 | 83.4 | 0.0 | 0.0 | 16.6 |
| OD-Cu +0.5 V | 0.989 | 71.9 | 0.0 | 23.2 | 4.8 | 95.2 |

Additional peak-position and fitting-procedure details for the Cu 2p and Cu LMM analyses are summarized in **Table S10**. Cu 2p positions are reported as representative maxima of the fitted component envelopes across the sample series, while Cu LMM positions are the fixed kinetic-energy reference centers used for the deconvolution.

**Table S10. Peak assignment and fitting constraints used for Cu 2p and Cu LMM analysis.** Cu 2p binding-energy values correspond to the main fitted component maxima observed across the spectra in **Figure S39**. Cu LMM kinetic-energy values correspond to the reference centers used for the fits in Figure S40. The Cu 2p analysis was interpreted together with Cu LMM because Cu0 and Cu+ overlap strongly in the Cu 2p region.

| **Analysis window** | **Fitted component** | **Assigned species** | **Peak position(s)** | **Fitting constraint / procedure** |
| --- | --- | --- | --- | --- |
| Cu 2p | Cu0 doublet | Metallic Cu / low-valent Cu | 2p3/2: 932.6-932.7 eV; 2p1/2: 952.3-952.4 eV | Fitted as part of the background-subtracted Cu 2p envelope; Cu^0^ and Cu^+^ are not separated using Cu 2p alone. |
| Cu 2p | Cu_2_O/Cu^+^ doublet | Cu^+^ / Cu_2_O-like Cu | 2p3/2: 932.1-932.2 eV; 2p1/2: 951.9-952.0 eV | Interpreted together with Cu LMM to quantify the low-valent Cu fraction. |
| Cu 2p | CuO/Cu^2+^ doublet | Cu^2+^ / CuO-like Cu | 2p3/2: 933.7-933.8 eV; 2p1/2: 953.5 eV | Included with Cu^2+^ shake-up satellites to constrain the Cu^2+^ oxide contribution. |
| Cu 2p | Cu(OH)_2_/Cu^2+^ doublet | Cu^2+^ hydroxide-like Cu | 2p3/2: 934.6-934.7 eV; 2p1/2: 954.4 eV | Used to track hydroxylated Cu^2+^ species formed during anodic conditioning. |
| Cu 2p | Cu^2+^ shake-up satellite envelope | Satellite features associated with Cu^2+^ | Main satellite: 942.6-944.0 eV; higher-BE satellite: 962.4-963.8 eV | Fitted as a combined satellite contribution; used to support Cu^2+^ assignment rather than as a separate Cu oxidation-state fraction. |
| Cu LMM | Cu(OH)_2_-like Cu^2+^ | Hydroxide-like Cu^2+^ | Kinetic energy: 916.25 eV | Reference center fixed; no individual peak-center movement allowed. |
| Cu LMM | Cu_2_O/Cu^+^ | Cu^+^ / Cu_2_O-like Cu | Kinetic energy: 916.99 eV | Reference center fixed; used to separate Cu^+^ from Cu^0^, which overlap in Cu 2p. |
| Cu LMM | CuO-like Cu^2+^ | Oxide-like Cu^2+^ | Kinetic energy: 917.57 eV | Reference center fixed; combined with Cu(OH)_2_-like Cu^2+^ for total Cu^2+^ contribution. |
| Cu LMM | Cu^0^ | Metallic Cu | Kinetic energy: 918.61 eV | Reference center fixed; relative component spacing fixed across spectra. |
| Cu LMM | Global fitting constraints | All Cu LMM components | Common FWHM: 3.12-3.80 eV; pseudo-Voigt eta = 0.90 | A common FWHM was used for each spectrum. A common spectrum-level kinetic-energy correction was allowed only for the anodically conditioned OD-Cu spectrum while keeping all relative component spacings fixed. Cu LMM fractions are treated as semi-quantitative because the envelopes overlap strongly. |

**Electrochemical study for Au-assisted Cu stabilization**

Electrochemical measurements provide additional evidence that Au modification changes the redox response of the Cu surface. In the MEA COR-a-HER configuration, AuCu exhibits a lower full-cell voltage than OD-Cu, PtCu, and AgCu at the same current density, indicating the most favorable apparent a-HER activity among the tested Cu-based anodes. CV measurements in 1 M KOH further show that OD-Cu develops more pronounced Cu redox/OH-related features and stronger hysteresis after electrolysis, whereas AuCu retains a more reproducible CV profile before and after operation. These trends are consistent with the spectroscopic evidence that Au modification suppresses the formation of strongly hydroxylated Cu species and stabilizes low-valent Cu under a-HER-relevant conditions. Similar connections between Cu valence, surface hydroxylation, and aldehyde oxidation/a-HER activity have been reported for Cu-based aldehyde oxidation systems.^30,49-52,56^

**Figure S27.** Cyclic voltammetry analysis of OD-Cu and AuCu before and after electrolysis.

Cyclic voltammograms of (a) OD-Cu before reaction, (b) OD-Cu after reaction, (c) AuCu before reaction, and (d) AuCu after reaction measured in 1 M KOH over five consecutive cycles. (e) Comparison of OD-Cu and AuCu before and after reaction. OD-Cu shows a more pronounced change in the Cu redox/OH adsorption region after electrolysis, whereas AuCu retains a more stable CV profile, supporting Au-assisted stabilization of the Cu surface during a-HER operation.

**Figure S28**. LSV comparison of OD-Cu and metal-modified Cu anodes for COR–a-HER operation.
Linear sweep voltammetry curves of the COR–a-HER MEA using OD-Cu, PtCu, AuCu, and AgCu as anode catalysts. The cathode was CuO & C-support_3, and the anolyte contained aldehyde reactant in alkaline electrolyte.

3. **Supplementary Note 3**

**Time-dependent analysis over oxidation states of OD-Cu and AuCu**

**Figure S29.** Raw data of full operando Raman spectroscopy of (a) OD-Cu and (b) AuCu foam under reducing conditions over time (s) and during the anodic hydrogen evolution reaction at various potentials (vs. RHE). The spectra have been normalized for clarity.

**Figure S30.** Operando Raman spectroscopy of (a) OD-Cu and (b) AuCu foam under reducing conditions over time (s) and during the anodic hydrogen evolution reaction at various potentials (vs. RHE). The spectra have been normalized, baseline subtracted and smoothed for clarity.

**Figure S31.** Deconvoluted Operando Raman spectra of (a) OD-Cu and (b) AuCu foam during the anodic hydrogen evolution reaction at 0.4 V (vs. RHE).

Operando Raman spectroscopy of OD-Cu electrodes reveals characteristic features associated with surface copper hydroxide species (either CuₓOHy or Cu–OH_ads) across a wide potential range^23-25^. These CuₓOH_y_-related bands rapidly diminish under cathodic bias (e.g., −0.1 V_RHE_), indicating reduction of the surface to metallic Cu. Upon reapplication of anodic potentials, the bands re-emerge, consistent with reversible surface oxidation. When the potential is switched from open circuit to anodic conditions, the CuₓOH_y_ bands shift to lower wavenumbers (e.g., from 523 to 548 cm⁻¹ and 624 to 603 cm⁻¹), likely due to electrochromic effects induced by the applied bias as confirmed from previous study^23^. Notably, the band at 603 cm⁻¹ increases with rising potential and extended time (**Figure 2m, 2n, S29, S30** and **S31**), after the deactivation of anodic hydrogen evolution (a-HER). This trend correlates with the oxygen evolution reaction, as confirmed by the increased potential (OER range) and O_2_ generation, suggesting the 603 cm⁻¹ band may be associated with OER. In contrast, the 548 cm⁻¹ peak remains relatively unchanged under the same conditions indicating peak at 603 cm^-1^ showing other chemical states than Cu_x_OH_y_ (peaks representing 548 cm-1 and 603 cm-1 should be paired with decreased or increased peak at the same time if they represent Cu_x_OH_y_^23,24^. Based on these observations and prior reports^26^, we assume the emergence of the 603 cm⁻¹ band to the formation of a Cu(III) oxyhydroxide (CuOOH), which may serve as a transient state involved in the oxygen evolution reaction (OER).

AuCu exhibits a persistent and broad Raman band (~480–650 cm⁻¹) under both reducing and oxidizing conditions. Even after 1 hour at −0.1 V_RHE, this oxide-related feature remains observable with a peak maximum near 546 cm⁻¹. Upon applying anodic potentials (0.1–0.5 V_RHE_), the band does not disappear but instead shifts toward higher wavenumbers (peaking at ~570 cm⁻¹) while remaining broad. This contrasts with the behavior of OD-Cu, where Cu–O(H) features typically vanish under reducing bias and reappear upon oxidation, suggesting that gold incorporation fundamentally alters the stability and redox response of copper-oxygen species on the surface.

Previous studies^27-29^, showed that a similarly broad Cu–O Raman band observed under anodic potentials originates from under-hydroxylated, disordered Cu–O surface frameworks rather than from bulk Cu(OH)₂. Isotopic substitution experiments (H₂O to D₂O) revealed that only a narrower ~525 cm⁻¹ Cu–OH peak shifts with deuteration, while the broader Cu–O band remains unchanged, supporting its assignment to non-hydroxyl Cu–O species. Notably, this Cu–O band persisted under anodic bias even as Cu–OH features diminished, and the process was reversible upon potential cycling. These results align with observations on Au–Cu foams, indicating that the broad band reflects a stable, low-coverage Cu–O phase that forms without full surface hydroxylation. This region includes modes characteristic of Cu–O vibrations^28^, and its persistence suggests that the surface copper remains in a partially oxidized, low-valent state (Cu⁺ or Cu^δ⁺) rather than converting fully to Cu⁰ or Cu²⁺ ^30^. Alloying with Au is known to suppress deep oxidation of Cu and may help stabilize such Cu–O species while lowering hydroxyl coverage^31,32^.

The broad peak at 301 cm⁻¹ observed for OD-Cu under reducing conditions is likely associated with Cu–O vibrational modes. Previous study have confirmed the origin of this peak using ¹³C-labeled and carbon-free electrolytes such as phosphate^25^. In contrast, AuCu did not exhibit a pronounced broad peak near 301 cm⁻¹, consistent with the results of linear combination fitting from XANES analysis, which indicate a higher proportion of CuO species in OD-Cu relative to AuCu (**Figure S32**).

**Figure S32.** In-situ X-ray absorption spectroscopy of (a) OD-Cu and (b) AuCu foam under reducing conditions in 0.5 M KOH electrolyte. The linear combination fitting of (c) OD-Cu and (d) AuCu was performed to track the oxidation states at various potentials.

4. **Supplementary Note 4**

**DFT Calculation framework.**

Density functional theory (DFT) calculations were performed using the Vienna *Ab Initio* Simulation Package (VASP) with the projector-augmented wave (PAW) method to describe electron-ion interactions within a frozen-core all-electron approach.^36,37^ To account for dispersion forces, Grimme’s correction was applied to the Perdew-Burke-Ernzerhof (PBE) functional.^38,39^ Structural relaxations were carried out using the conjugate gradient method, with atomic positions optimized along the Hellmann-Feynman force direction until the residual force on each atom was below 0.001 eVÅ⁻¹. A plane-wave energy cutoff of 520 eV ensured numerical accuracy. The crystal structures of copper (Cu) (Fm$\bar{3}m$ space group) and cuprous oxide (Cu₂O) (Pn$\bar{3}m$ space group) were constructed using the Atomic Simulation Environment (ASE).^31^ Using the optimized bulk structures of Cu and Cu₂O, we generated the Cu(111) and Cu₂O(111) surfaces using ASE. Adsorption energies were evaluated on four-layered surfaces constructed with a 5×5 supercell of Cu(111), Cu₂O(111), oxygen-deficient Cu (OD-Cu, derived from Cu₂O with Cu⁰-Cu⁺ oxidation states), Au-modified Cu (AuCu derived from OD-Cu by Au substitution), and hydroxylated Cu (Cu(OH)_ads_). Au doping was incorporated by substituting Cu atoms on the top layer of the OD-Cu, revealing a thermodynamic preference for Au occupation at Cu⁰ sites. A high concentration mv AuCu system was constructed within a random alloy substitution framework, generating 50 unique configurations. To systematically identify the most stable structures, a Hausdorff distance filtering algorithm was employed, enabling the removal of redundant configurations. The algorithm iteratively compared newly generated atomic structures against previously selected configurations, retaining only those exceeding a predefined Hausdorff distance threshold of 0.5 Å. This approach ensured a diverse structural dataset while minimizing computational cost. **Figure S33** presents the most stable configurations of Au-doped Cu at both low and high doping concentrations. Periodic boundary conditions were applied in the *xy* plane, while a 30 Å vacuum region was introduced along the *z*-axis to eliminate spurious interactions between periodic images. A 3×3×1 *k*-point grid was used for Brillouin zone sampling, ensuring convergence of electronic states. Adsorption energies were computed to quantify the interaction strength of the furfural (FF) molecule on the designed catalytic surfaces. The adsorption energy of FF molecule is calculated as follows:

$$\Delta_{f}G\left( i \right)=\mu_{FF@*}-\mu{- \mu}_{\mathrm{FF}}+\Delta E_{ZPE,i}+\Delta H_{i}\left( 0\to300K \right)-T\Delta S_{i}$$

(S1)

The adsorption energy of furfural (FF) on the studied surfaces is defined in terms of the ground-state energies of the adsorbed system $(\mu_{FF@*})$, the clean surface (μ), and the gas-phase furfural molecule$(\mu_{\mathrm{FF}})$. Finite-temperature corrections, including zero-point energy $\left( \Delta E_{ZPE,i} \right),$enthalpy $(\Delta H_{i}\left( 0\to300K \right))$, and entropy $(T\Delta S_{i})$ were incorporated to refine the energy calculations. These corrections were determined at room temperature and standard atmospheric pressure (P = 1 atm) within the harmonic approximation, as implemented in the Atomic Simulation Environment (ASE) and VASPKIT.^39,40^ To account for solvation effects, an implicit Poisson–Boltzmann solvation model was employed using vaspsol*++*, with the dielectric constant of water set to 80 to approximate an aqueous environment.^42^

To understand why Cu₂O(111) in alkaline electrolytes preferentially transforms into Cu(OH)_ads_ while the AuCu surface remains more stable, we employed two distinct approaches: (i) analyzing the behavior of (OH)_ads_ across various charge states and (ii) investigating the water interface interactions with both systems. The first approach demonstrates that (OH)_ads_ becomes more stabilized on Cu₂O(111) as the surface charge concentration increases (**Figure 3a**). Additionally, the ΔG of (OH)_ads_ on Cu₂O(111) becomes more favorable in alkaline media, further enhancing surface stabilization (**Figure 3b**). In the second approach, we performed ab-initio molecular dynamics (AIMD) simulations at room temperature to explore interfacial interactions with water. The structural evolution and bond length fluctuations were analyzed over a 10 ps trajectory **(Figure S34** and **S35)** to quantify the stability of Cu-O interactions in both systems. To ensure a rigorous assessment, we distinguished surface O atoms, which are coordinated to at least two Cu atoms in Cu₂O, from water O atoms, which belong to stable H₂O molecules bonded to two hydrogen atoms. Bond distances between Cu and water O atoms were computed using a 2.5 Å cutoff, capturing only meaningful interactions. The calculations reveal that Cu₂O(111) exhibits strong interactions with water, with Cu-O bond lengths fluctuating between 1.87 and 2.0 Å, promoting surface hydroxylation and leading to its transformation into Cu(OH)_ads_. Conversely, on the AuCu surface, Cu-O bond lengths range from 2.1 to 2.4 Å, indicating weaker interactions that suppress hydroxylation and stabilize the surface. These findings provide key insights into the catalytic behavior of both systems: Cu₂O(111) exhibits high activity toward the oxygen evolution reaction (OER), while AuCu preferentially facilitates FFOR. These differences highlight the role of surface electronic structure and metal oxidation state in governing interfacial stability under alkaline conditions. AIMD simulations were conducted within the canonical ensemble (NVT), employing the Nosé thermostat for temperature control, with a 1 fs time step over 10 ps.^38^ The equations of motion were integrated using the Verlet algorithm, and Γ-point sampling was applied to ensure computational efficiency.

The thermodynamic mechanism of the furfural oxidation reaction (FFOR) was investigated by constructing free energy profiles for each system using Eq. S1. The most stable configurations along the FFOR reaction pathway are presented in **Figures S36, S37,** and **S38** for all considered systems**.** We conducted extensive DFT calculations to understand the furfural adsorption dependence on the d-band center of Cu active sites. The Hammer and Nørskov d-band model is employed to calculate the d-band center of the Cu on all considered systems.^38,40^ This parameter is a crucial descriptor in predicting the catalytic activity of the catalyst. In our analysis, the summation of all projected d-states is approximated by the d-band center, which is denoted as$\varepsilon_{d}$:

$\varepsilon_{d}=\frac{\int_{{-E}_{min}}^{E_{max}} n_{d}(\varepsilon)\varepsilon d\varepsilon}{\int_{{-E}_{min}}^{E_{max}} n_{d}(\varepsilon)d\varepsilon}$,

(S2)

where the projected density of states and energy of d-states are represented by *n*_d_ and *ε,* respectively. The energy limits $E_{\min},$and$E_{\max}$ chosen to include the relevant range of density of states associated with d-states of the system. The projected density of d-states for all systems and specific energy ranges used for each system are indicated in **Figure S39**, where appropriate values of EMIN, EMAX, and a sufficiently large NEDOS were selected during the calculations to resolve peak fluctuations in the density of states.

We performed non-spin-polarized DFT calculations to investigate the electronic and thermodynamic basis for the observed CO reservoir effect. In alignment with our experimental characterization, we examined two representative surface models: the pristine CuO(111) surface and a carbon-modified CuO(111)/C heterostructure (**Figure S40**). In the latter, the carbon layer serves as a molecular-scale proxy for the porous carbon-copper interface observed experimentally. This model specifically probes the synergistic interactions at the catalyst-support boundary, where the carbon atoms directly bonded to the copper surface act as potential local storage sites for CO molecules, mimicking the confinement effects within the porous C-support_3 architecture. Our results show that CO molecules are significantly stabilized at carbon sites directly bonded to Copper, reaching an adsorption energy minimum. While CO adsorption on bare CuO(111) shows a continuous increase in stabilization that can lead to surface saturation and site-blocking, the CuO/C interface provides a unique energetic landscape where CO is effectively trapped at the interface. This theoretical potential well provides a thermodynamic basis for the high τ₀ observed in Electrochemical impedance spectroscopy (EIS), confirming that the porous carbon support functions not merely as a physical spacer, but as a chemical reservoir that sustains local CO availability even under the low partial pressures (0.25 atm) where the bare CuO surface would otherwise be dominated by the hydrogen evolution reaction.

**Figure S33.** The chemical structures of the investigated surfaces include (a) pristine Cu(111), (b) Cu₂O(111), (c) Cu(OH)_ads_, (d) the OD-Cu surface, which is generated by introducing oxygen vacancies into Cu₂O(111), resulting in the coexistence of Cu⁺ and Cu⁰ sites, and (e and f) the AuCu surface at both low and 10% doping concentrations. The yellow, brown, red, and white spheres correspond to Au, Cu, O, and H atoms, respectively.

**Figure S34.** The total energy fluctuations were computed at room temperature using ab initio molecular dynamics (MD) simulations for water molecules interacting with the top-layer surface atoms of (a) Cu₂O(111) and (b) AuCu (AuCu consist of Cu⁰-Cu⁺ oxidation sates) surface. The inset shows the chemical structure of solid/water interface for both systems. The yellow, brown, red, and white spheres correspond to Au, Cu, O, and H atoms, respectively.

**Figure S35.** The average fluctuations in Cu–O bond length were computed at room temperature using ab initio molecular dynamics (MD) simulations for water molecules interacting with the top-layer surface atoms of (a) Cu₂O(111) and (b) AuCu (AuCu consist of Cu⁰-Cu⁺ oxidation sates) surface. The inset shows the chemical structure of solid/water interface. The brown, red, and white spheres correspond to Cu, O, and H atoms, respectively.

**Figure S36.** The most stable configurations of key reaction intermediates involved in the furfural oxidation reaction mechanism on the Cu (111) are presented, providing insights into the reaction pathway. The brown, grey, red, and white spheres correspond to Cu, C, O, and H atoms, respectively.

**Figure S37.** The most stable configurations of key reaction intermediates involved in the furfural oxidation reaction mechanism on OD-Cu (Cu consist of Cu⁰-Cu⁺ oxidation sates) surface are presented, providing insights into the reaction pathway. The brown, grey, red, and white spheres correspond to Cu, C, O, and H atoms, respectively.

**Figure S38.** The most stable configurations of key reaction intermediates involved in the furfural oxidation reaction mechanism on AuCu (AuCu consist of Cu⁰-Cu⁺ oxidation sates) surface are presented, providing insights into the reaction pathway. The yellow, brown, grey, red, and white spheres correspond to Au, Cu, C, O, and H atoms, respectively.

**Figure S39.** Projected density of states (PDOS) of Cu surface d-orbitals for (a) Cu(111), (b) OD-Cu, and (c) AuCu systems, computed using the PBE functional with D3 dispersion corrections.

**Figure S40.** *CO adsorption energy on pristine CuO(111) and CuO(111)/C support catalyst. The inset shows the chemical structure of *CO most stable configurations on CuO(111) and CuO(111)/C support. The brown, grey, red, and white spheres correspond to Cu, C, O, and H atoms, respectively.

5. **Supplementary Note 5**

**Energy demand analysis**

**Methods and assumptions**

For the analysis of the energy penalty and techno-economic feasibility, we have selected benchmark systems from the literature. These systems exhibit a current density exceeding 200 mA/cm^2^: neutral CO_2_R, acidic CO_2_R, and cascade CO_2_-to-CO & COR. These selections prioritize high carbon efficiency, aiming to minimize CO_2_ loss into the electrolyte. The energy cost of producing 1 ton of ethylene was analyzed by calculating energy costs of electricity (all systems), heating (SOEC part in cascade CO_2_ to CO & COR) and product separation (all systems). For SOEC parameter, we used full cell potential (V) of 1.06, CO faradaic efficiency (%) of 95, total current density (mA/cm^2^) of 815, single-pass conversion (%) of 55 % and operating temperature of (^0^C) of 800 based on previous report.^7,8^ For COR & a-HER system, we used parameters we used full cell potential (V) of 0.92, ethylene faradaic efficiency (%) of 63, total current density (mA/cm^2^) of 400 and single-pass conversion (%) of 92 %.

## **Energy summary**

## **Heating (SOEC only)**

Previous reports^7^ standards for calculating the energy required for heating the nichrome component and CO_2_ reactant gas. Same approaches are applied here.

First, we need to area of SOEC electrolyser for the nichrome heating

$$\begin{aligned} Area of electrolyser [m^{2}] =\frac{{Total current needed}_{SOEC} [mA]}{{Current density}_{{CO}_{2}, SOEC}[\frac{mA}{{cm}^{2}}]}*10000 {cm}^{2}/m^{2} \end{aligned}$$

The total current required for SOEC can be calculated as follows,

$${Total current needed}_{SOEC} \left[ A \right]=\frac{CO required rate \left[ \frac{mol}{s} \right]*number of e^{-}transferred for CO*Faradaic constant[\frac{A s}{C}]}{{FE}_{CO, SOEC}}$$

and we can calculate the CO required rate from COR data:

$$CO required rate \left[ \frac{mol}{s} \right]= \frac{{Total current needed}_{COR}[A]}{Faradaic constant [\frac{A s}{C}]}*\sum_{i} \frac{{FE}_{{product}_{i}}*Molar ratio of {product}_{i}}{number of e^{-} transferred {product}_{i}}$$

$${Total current needed}_{COR} \left[ A \right]=\frac{Ethylene production rate \left[ \frac{mol}{s} \right]*Number of e^{-}transferred for ethylene*Faradaic constant[\frac{A s}{C}]}{{FE}_{ethylene}}$$

$$Ethylene production rate \left[ \frac{mol}{s} \right]=\frac{Ethylene production [\frac{g}{day}]}{{MW}_{ethylene}\left[ \frac{g}{mol} \right]*86400[\frac{s}{day}]}=0.413 \frac{mol}{s}$$

Assuming electrolyser thickness is 5 cm

$$\begin{aligned} Mass of nichrome \left[ kg \right] =Thickness\left[ m \right]*Area of electrolyzer [m^{2}]*Density of nicrhome[\frac{kg}{m^{3}}]\# \end{aligned}$$

$$\begin{aligned} Heating energy for nichrome [\frac{GJ}{tonne CO}] =Nichrmoe heat capacity[\frac{J}{kg*K}]*\Delta T \left[ K \right]*Mass of nichrome[kg] \\ \end{aligned}$$

Where the heating energy required from CO_2_ is

$$\begin{aligned} Heating energy for {CO}_{2} [\frac{GJ}{tonne CO}] ={CO}_{2} heat capacity \left[ \frac{J}{Kg*K} \right]*\Delta T [K]*mass of {CO}_{2}[kg] \\ \end{aligned}$$

The mass of CO_2_ can be calculated as follows:

$$mass of {CO}_{2}\left[ kg \right]= \frac{CO required per day \left[ kg \right]*{MW}_{{CO}_{2}}}{{MW}_{CO}}*\frac{1}{{SPCE}_{CO}}$$

And we can calculate the CO requirement from FE data of each cases.

$$CO required per day \left[ \frac{kg}{day} \right]= \frac{{Total current needed}_{COR} [A]}{Faradaic constant[\frac{A s}{C}]}*\sum_{i} \frac{{FE}_{{product}_{i}}*Molar ratio of {product}_{i}}{number of e^{-} transferred {product}_{i}}*{MW}_{CO}*86400 \frac{s}{day}*\frac{1 kg}{{10}^{3} g}$$

Since 2 mol of CO are required to produce 1 mol of ethylene, the total energy demand of SOEC heating is

$$Heating SOEC [\frac{GJ}{tonne ethylene} ]=\left\{ Heating energy for {CO}_{2} \left[ \frac{GJ}{tonne CO} \right]+Heating energy for nichrome \left[ \frac{GJ}{tonne CO} \right] \right\}*2[\frac{tonne CO}{tonne ethylene}]$$

## **Electricity**

For the SOEC part, electrolyser electricity can be calculated as follows

$${Electricity}_{SOEC}[\frac{GJ}{tonne ethylene}]= \frac{{Total current needed}_{SOEC} \left[ A \right]*{Cell potential}_{SOEC}[V]}{CO required per day [tonne CO]}*24\left[ \frac{hours}{day} \right]*0.0036\left[ \frac{GJ}{kWh} \right]*\frac{1 tonne CO}{1 tonne ethylene}$$

Total current needed for SOEC and the amount of CO required per day was calculated in previous section.

For the COR part, electrolyser electricity can be calculated as follows

$${Electricity}_{COR}[\frac{GJ}{tonne ethylene}]= \frac{{Total current needed per day}_{COR} \left[ A \right]*{Cell potential}_{COR}[V]}{CO required per day [tonne CO]}*24\left[ \frac{hours}{day} \right]*0.0036\left[ \frac{GJ}{kWh} \right]*\frac{1 tonne CO}{1 tonne ethylene}$$

## **Cathode gas product separation**

To calculate the cathode gas product separation cost, we need to consider the single pass conversion efficiency (SPCE) of the SOEC and COR parts. To separate unreacted CO_2_ or CO from product gas stream, pressure swing adsorption (PSA) is used for the calculation in this section.^7^ Specifically, this will be equal to the cost of separating the unreacted CO_2_ from the product stream of the SOEC and the cost of separating the unreacted CO from the product stream of the CORR.

For the SOEC, the CO outlet flow rate is

$$\begin{aligned} CO outlet flow \left[ \frac{m^{3}}{h} \right]=\frac{CO amount required per day[\frac{tonne}{day}]*Gas constant\left[ \frac{J}{mol*K} \right]*Temperature[K]}{Molecular weight of product[\frac{g}{mol}]*Pressure[Pa]} \\ \end{aligned}$$

For the CO amount required per day,

$$CO amount required per day \left[ \frac{tonne}{day} \right]=\frac{{Total current needed}_{COR} [A]}{Faradaic constant}*\sum_{i} \frac{{FE}_{{product}_{i}}*Molar ratio of {product}_{i}}{number of e^{-} transferred {product}_{i}}*{MW}_{CO}*86400 \frac{s}{day}*\frac{1 tonne}{{10}^{6} g}$$

The unreacted CO_2_ flow at the SOEC outlet based on 55% CO_2_ SPCE is

$$\begin{aligned} Unreacted {CO}_{2} outlet flow \left[ \frac{m^{3}}{h} \right]=\frac{CO outlet flow[\frac{m^{3}}{h}]*mole ratio of {CO}_{2} to CO*\left( \frac{1-{SPCE}_{SOEC}\left[ decimal \right]}{1} \right)}{{SPCE}_{SOEC}\left[ decimal \right]} \\ \end{aligned}$$

The cathode separation energy demand for the SOEC part is

$$Total product flow rate of the SOEC\left[ \frac{m^{3}}{h} \right]= CO outlet flow \left[ \frac{m^{3}}{h} \right]+ Unreacted {CO}_{2} outlet flow \left[ \frac{m^{3}}{h} \right]$$

$$Gas separation of the SOEC part\left[ \frac{GJ}{tonne C_{2}H_{4}} \right]= \frac{PSA energy demand \left[ \frac{kWh}{m^{3}} \right]*Total product flow rate}{CO amount required per day \left[ \frac{tonne}{day} \right]}*\frac{{tonne}_{CO}}{{tonne}_{C2H4}}$$

The cathode separation energy demand for the COR part is

$$Ethylene flow rate \left[ \frac{m^{3}}{h} \right]=\frac{Ethylene production per day\left[ \frac{tonne}{day} \right]*{10}^{6}\left[ \frac{g}{tonne} \right]*Gas constant\left[ \frac{J}{mol*K} \right]*Temperature[K]}{Molecular weight of ethylene [\frac{g}{mol}]*Pressure \left[ Pa \right]*24 [\frac{h}{day}]}$$

$$H_{2} flow rate\left[ \frac{m^{3}}{h} \right]= \frac{Total current [A]*\left( 1-FE\left[ decimal \right] \right)*Gas constant\left[ \frac{J}{mol*K} \right]*Temperature[K]}{number of e^{-} transferred to hydrogen*Faraday constant\left[ \frac{A*s}{C} \right]*Pressure[Pa]}$$

$$Unreacted CO outlet flow \left[ \frac{m^{3}}{h} \right]=\frac{Ethylene flow [\frac{m^{3}}{h}]*mole ratio of CO to ethylene*\left( \frac{1-{SPCE}_{COR}\left[ decimal \right]}{1} \right)}{{SPCE}_{COR}\left[ decimal \right]}$$

$$Total product flow rate of the COR\left[ \frac{m^{3}}{h} \right]=H_{2} flow rate\left[ \frac{m^{3}}{h} \right]+Ethylene flow rate\left[ \frac{m^{3}}{h} \right]+Unreacted CO flow rate\left[ \frac{m^{3}}{h} \right]$$

$Gas separation of the COR part\left[ \frac{GJ}{tonne C_{2}H_{4}} \right]=\frac{PSA energy demand \left[ \frac{kWh}{m^{3}} \right]*Total product flow rate [\frac{m^{3}}{h}]}{Ethylene production per day \left[ \frac{tonne}{day} \right]}$

Following data is energy cost summary comparison between this work and previous reports.

**Table S11.** Energy cost summary comparison between this work and selected literature systems.

| **Parameter / System** | **Ref. 8** | **Ref. 9** | **Ref. 10** | **Ref. 11** | **This work** |
| --- | --- | --- | --- | --- | --- |
| Full cell potential (V) | 2.1 | 2.32 | 1.45 | 2.3 | 0.92 |
| Ethylene Faradaic efficiency (%) | 40 | 40 | 50 | 84 | 63 |
| Total current density (mA cm⁻²) | 230 | 144 | 180 | 320 | 400 |
| Single-pass conversion (%) | 90 | 5 | 5 | 20 | 91 |
| **Energy demand (GJ tonneC₂H₄⁻¹)** |  |  |  |  |  |
| Electrolyzer electricity | 144.5 | 159.6 | 79.8 | 75.3 | 40.2 |
| Cathode separation | 1.1 | 56.5 | 35.7 | 8.9 | 1.1 |
| Anode separation | - | - | - | - | - |
| CO₂-to-CO SOEC energy demand | 28.1 | 28.1 | 28.1 | 28.1 | 28.1 |
| **Overall energy demand** | **173.7** | **244.2** | **143.7** | **112.3** | **69.4** |

Note: The upstream CO₂-to-CO SOEC energy demand is included for all cases to enable comparison on a CO₂-to-ethylene basis.

6. **Supplementary Note 2**

**Techno-economic analysis (TEA) over COR-a HER system**

**Methods and assumptions**

To confirm the economic potential of COR-a HER system using renewable electricity, TEA was performed based on the modified model from previous reports.^12-14^ **Table S12**, **S13** and **S14** shows the parameter that we used to calculate the plant-gate levelized cost for the generation of one tonne of ethylene and the corresponding production of ethanol, ethylene, acetate, hydrogen and furoic acid. The costs for the eletrolyser, catalyst, membrane, installation, input chemicals, electricity, balance of plant, gas and liquid product separation and other operating expense were considered in this TEA (**Table S12**). Here, we used the performance from cathode part using copper oxide with C-support_3 and anode part using AuCu for a-HER and commercial iridium oxide for OER. The following performance value was used for calculation which is based on experimental values indicated in **Table** **S12**.

**Table S12**. Performance parameter for comparison between COR-OER and COR-aHER

|  | Cathode products FE (%) | | | | | | Anode products FE (%) | | | Cell voltage | | Current density |
| --- | --- | --- | --- | --- | --- | --- | --- | --- | --- | --- | --- | --- |
|  | Hydrogen | Ethylene | Acetate | Ethanol | Propanol | Hydrogen | | Furoic acid | V | | mA/cm^2^ | |
| OER | 8 | 63 | 8 | 13 | 3 | - | | - | 2.51 | | 400 | |
| a-HER | 8 | 63 | 8 | 13 | 3 | 93 | | 95 | 0.92 | | 400 | |

**Table S13**. Calculation factors for TEA sensitivity analysis based on previous reports.^12-14^

|  | Optimistic | Average | Conservative |
| --- | --- | --- | --- |
| Electrolyser factor ($/kW) | 250 | 550 | 700 |
| Capacity factor | 0.85 | 0.8 | 0.75 |
| CRF for electrolyser | 0.081 | 0.086 | 0.094 |
| Electrolyser lifetime (year) | 30 | 25 | 20 |
| CRF for membrane and catalyst | 0.19 | 0.24 | 0.38 |
| Catalyst and membrane lifetime (year) | 7 | 5 | 3 |
| CRF electrolyte | 1.07 | 1.41 | 2.10 |
| Electrolyte lifetime (month) | 12 | 9 | 6 |
| Electricity ($/kWh) | 0.03 | 0.04 | 0.05 |
| Operating cost factor |  |  |  |
| Labor | 0.1 | 0.11 | 0.12 |
| Maintenance | 0.02 | 0.03 | 0.04 |
| Taxes and insurance | 0.04 | 0.05 | 0.06 |
| Depriciation | 0.07 | 0.08 | 0.09 |
| Miscellaneous overheads | 0.2 | 0.3 | 0.4 |

**Table S14**. Input and output chemical costs for TEA sensitivity analysis

| Reactant chemicals (Optimistic / Average / Conservative) | | Product chemicals | |
| --- | --- | --- | --- |
| Input chemical costs ($/ton) |  | **Product chemical costs ($/ton)** | |
| Carbon monoxide^7,12^ | 300 | Hydrogen^12-14^ | 1900 |
| Water^12^ | 5 | Ethylene^12-14^ | 1300 |
| Potassium hydroxide^14^ | 800 / 1000 / 1280 | Acetate^12-14^ | 595 |
| Sulfuric acid^7,14^ | 20 / 40 / 60 | Ethanol^12-14^ | 1000 |
| Furfural^7,14^ | 1170 | Propanol^12-14^ | 1430 |
|  |  | Furoic acid^15,21^ | 6230 |
|  |  | Furfuryl alcohol^15,21^ | 1250 |

**Capital expenditures (CAPEX) for producing 100 tonne_ethylene_ / day**

In Capital expenditures, we considered gas and liquid separation plant capital costs, balance of plants, installation costs, electrolyzer, membrane and catalyst costs. For gas and liquid separation, we used PSA and liquid distillation method.

1. Calculating the electrolyzer, membrane and catalyst costs

$$Total electrolyzer cost \left( \$ \right)=Power consumed \left[ kW \right]*Electrolyzer factor \left[ \frac{\$}{kWh} \right]*\frac{Reference current density [\frac{mA}{{cm}^{2}}]}{Input current density [\frac{mA}{{cm}^{2}}]}$$

$$Electrolyzer cost \left[ \frac{\$}{tonne ethylene} \right]=\frac{{CRF}_{electrolyzer}*Total electrolyzer cost [\$]}{Capacity factor*365 \left[ \frac{day}{year} \right]*Production [\frac{tonne ethylene}{day}]}$$

Toal one-time costs for catalyst and membrane was assumed as 5 % of the electrolyzer.

$$Catalyst and membrane cost \left[ \frac{\$}{tonne ethylene} \right]=\frac{{CRF}_{catalyst and memrbane}*0.05*Total electrolyzer cost [\$]}{Capacity factor*365 \left[ \frac{day}{year} \right]*Production [\frac{tonne ethylene}{day}]}$$

2. Calculating PSA capital cost

To calculate gas separation capital cost, we followed the PSA model for bio-upgrading.^13^ The reference used a cost of $1989043 with a flowrate capacity of 1000 m^3^h^-1^, scaling factor of 0.7 and an energy factor of 0.25 kWh m^-3^.^8,14^

$$PSA captital cost \left[ \$ \right]=\$1989043* \left[ \frac{Total product flow}{1000 m^{3}h^{-1}} \right]^{0.7}$$

$$PSA capital cost \left[ \frac{\$}{tonne ethylene} \right]=\frac{{CRF}_{electrolyzer}*PSA captial cost [\$]}{Capacity factor*365 \left[ \frac{day}{year} \right]*production [\frac{tonne ethylene}{day}]}$$

And total gas product flow can be calculated as

$$Total product flow rate of the COR \left[ \frac{m^{3}}{h} \right]=H_{2} flow rate\left[ \frac{m^{3}}{h} \right]+Ethylene flow rate\left[ \frac{m^{3}}{h} \right]+Unreacted CO flow rate[\frac{m^{3}}{h}]$$

$$Ethylene flow rate [\frac{m^{3}}{h}]=\frac{Ethylene production [\frac{tonne}{day}]*Gas constant \left[ \frac{J}{mol K} \right]*Temperature [K]}{Molecular weight of ethylene [\frac{g}{mol}]*Pressure [Pa]}$$

$$H_{2} flow rate [\frac{m^{3}}{h}]= \frac{Total current [A]*\left( 1-FE\left[ decimal \right] \right)*Gas constant \left[ \frac{J}{mol K} \right]*Temperature[K]}{number of e^{-} transferred for hydrogen*Faraday constant \left[ \frac{A s}{C} \right]*Pressure [Pa]}$$

$$Unreacted CO outlet flow [\frac{m^{3}}{h}]=\frac{Ethylene flow rate [\frac{m^{3}}{h}]*mole ratio of CO to ethylene*\left( \frac{1-{SPCE}_{COR}\left[ decimal \right]}{1} \right)}{{SPCE}_{COR}\left[ decimal \right]}$$

3. Calculating distillation capital cost

For liquid separation, we used distillation model targeting ethanol, propanol, acetate, furfuryl alcohol and furoic acid as product. We assumed that catholyte and anolyte are both circulated until the concentration of product reaches 10%. Based on previous result,^14^ the distillation capital reference cost for a flow rate capacity 1000 L min^-1^ are $4687910 and $ 4162240 for n-propanol and ethanol, respectively. The distillation operating reference cost of n-propanol and ethanol are $ 17079 and $ 10543. Furoic acid can be readily separated through facile precipitation under highly alkaline conditions, resulting in the formation of potassium furoate. This occurs because its water solubility is less than 3%.^12,14^ For simplicity of calculation, we use the parameters of n-propanol above for the calculation of all liquid product separation since n-propanol has a highest cost of distillation among the liquid products.

$$Distillation capital cost \left[ \$ \right]=\$4687910*{(\frac{Aqueous solution flowrate [\frac{L}{min}]}{1000 \frac{L}{min}})}^{0.7}$$

$$Distillation capital cost \left[ \frac{\$}{tonne ethylene} \right]=\frac{{CRF}_{electrolyzer}*Distillation captial cost [\$]}{Capacity factor*365 [\frac{day}{year}]*production [\frac{tonne ethylene}{day}]}$$

And we can get aqueous solution flow rate by

$$Aqueous solution flowrate \left[ \frac{L}{min} \right]=\frac{Total product flowrate [\frac{L}{min}]}{Product concentration \left[ decimal \right]}$$

$$Total product flow rate \left[ \frac{L}{min} \right]=\sum Product flow rate\left[ \frac{L}{min} \right]= \sum\frac{Product production rate [\frac{kg product}{day}]*1000 [\frac{L}{m^{3}}]}{Product density [\frac{kg}{m^{3}}]*24 [\frac{hours}{day}]*60[\frac{min}{hours}]}$$

Product production rate can be derived from faradaic efficiency equation, which is

$$Product production rate\left[ \frac{kg product}{day} \right]=Ethylene production rate \left[ \frac{kg product}{day} \right]*\frac{{MW}_{product}*{number of e^{-}}_{ethylene}*FE of ethylene}{{MW}_{ethylene}*{number of e^{-}}_{product}*FE of product}$$

4. Installation cost and balance of plant (BOP)

$$Instllation cost\left[ \frac{\$}{tonne ethyelne} \right]=Lang factor*Total capital cost\left( PSA captial cost+Distillation captial cost+electrolyzer cost+catalyst and membrane cost \right)\left[ \frac{\$}{tonne ethyelne} \right]$$

$$Balance of palnt\left[ \frac{\$}{tonne ethyelne} \right]=Balance of plant factor*Total capital cost\left[ \frac{\$}{tonne ethyelne} \right]$$

Balance of plant factor and Lang factor are 0.5 and 1 based on previous reports.^7-9^

**Operating expenditures (OPEX) for producing 100 tonne_ethylene_ / day**

In operating expenditure, we considered distillation operating costs, PSA operating costs, electricity, input chemicals and other operating cost.

1. Electricity costs

$$Electricity cost \left[ \frac{\$}{tonne ethylene} \right]=\frac{Power consumed \left[ kW \right]*24 \left[ \frac{hours}{day} \right]*electricity price [\frac{\$}{kWh}]}{Production rate [\frac{tonne ethylene}{day}]}$$

2. PSA and distillation operating costs

We used an energy consumption rate of 0.25 kWh/m³ for PSA operating costs, derived from prior studies.

$$PSA operating cost \left[ \frac{\$}{tonne ethylene} \right]=\frac{0.25 [\frac{kWh}{m^{3}}]*Total gas flow rate [\frac{m^{3}}{hour}]*24 [\frac{hours}{day}]*electricity price[\frac{\$}{kWh}]}{Production rate [\frac{tonne ethylene}{day}]}$$

Referring to the operational cost data from propanol distillation, we opted for parameters based on n-propanol for calculating the separation costs of all liquid products. This choice accounts for the higher distillation costs associated with n-propanol, ensuring a more conservative estimate.

$$Distillation operating cost \left[ \frac{\$}{tonne ethylene} \right]=\frac{Aqueous solution flow rate [\frac{L}{min}]*17078.9 [\frac{\$}{day}]}{1000\frac{L}{min}*Production rate [\frac{tonne ethylene}{day}]}$$

3. Input chemical costs

The expenses associated with chemicals or reactants are essential for the COR and a-HER reactions, targeting the production of 100 tons of ethylene per day, were examined. The prices for these chemicals are outlined in **Table S9**, drawing from previous research findings. Additionally, for the a-HER reaction, we factored in the consumption of KOH and H_2_SO_4_ in the deprotonation of furoic acid to furoate and the subsequent regeneration of furoate to furoic acid.

In the COR part, the expenses related to water consumption, electrolyte, and CO are taken into account. The calculation methodology is outlined as follows.

The cost of consumed water is

$$Cost of consumed water \left[ \frac{\$}{tonne ethylene} \right]= \frac{Total current needed \left[ A \right]*86400\left[ \frac{s}{day} \right]*{MW}_{water}\left[ \frac{kg}{mol} \right]}{\# of electron transfered per water*Faradaic constant\left[ \frac{C}{mol} \right]*1000}*\frac{Water price [\frac{\$}{tonne H_{2}O}]}{Production rate [\frac{tonne ethylene}{day}]}$$

The cost of electrolyte is

$$Cost of electrolyte \left[ \frac{\$}{tonne ethylene} \right]=\frac{{CRF}_{electrolyte}*Cost of electrolyte [\$]}{Capacity factor*365[\frac{day}{year}]*Production rate [\frac{tonne ethylene}{day}]}$$

Where cost of electrolyte [$] can be calculated as

$$Cost of electrolyte \left[ \$ \right]=Volume of electrolyte \left[ L \right]*{MW}_{KOH}\left[ \frac{kg}{mol} \right]*1\frac{mol}{L}*KOH price\left[ \frac{\$}{kg} \right]+Volume of electrolyte \left[ L \right]*Water price \left[ \frac{\$}{kg} \right]$$

Based on our laboratory-scale data, we presumed a constant volume of 100 liters of electrolyte required per square meter of electrolyzer. Hence, the total volume of electrolyte required is calculated as follows:

$$Volume of electrolyte \left[ L \right]=\frac{Total current needed[A]}{Current density[\frac{mA}{{cm}^{2}}]*{(\frac{100cm}{1m})}^{2}}*100\frac{L}{m^{2}}$$

The cost calculation for CO is based on the quantity utilized in the COR process. The calculation is as follows:

$$Cost of CO\left[ \frac{\$}{tonne ethylene} \right]=[\sum_{i} Molar ratio \left[ \frac{CO}{{Product}_{i}} \right]*\frac{{Product}_{i} production amount [\frac{g}{day}]}{{MW}_{i} \left[ \frac{g}{mol} \right]}]*\frac{{MW}_{CO}}{100 \frac{tonne ethylene}{day}}* CO price [\frac{\$}{tonne}]$$

In the a-HER section, we consider the costs associated with water consumption, electrolyte, furfural, potassium hydroxide, and sulfuric acid. However, it's important to note that furfural undergoes the Cannizzaro reaction under alkaline conditions, yielding furfuryl alcohol and furoic acid in a 1:1 ratio. Therefore, we also account for the simultaneous occurrence of the Cannizzaro reaction and a-HER (furfural oxidation to furoic acid only) in this calculation, with a reaction ratio between a-HER and the Cannizzaro reaction set at 2:1 based on experimental data at 400 mA/cm^2^.

$$2CO+4H_{2}O+8e^{-}\to C_{2}H_{4}+8{OH}^{-}$$

$$8C_{4}H_{3}OCHO+8{OH}^{-}\to8C_{4}H_{3}OCOOH+4H_{2}+8e^{-}$$

$${4C}_{4}H_{3}OCHO \to{4C}_{4}H_{3}OCOOH+ {4C}_{4}H_{3}OCH_{2}OH$$

$$C_{4}H_{3}OCOOH+KO H\to{4C}_{4}H_{3}OCOOK+H_{2}O$$

First, we need to determine furoic acid production from a-HER reaction. The calculation methodology is outlined as follows:

$$Fuoric acid production from anodic HER [\frac{tonne}{day}]= \frac{Furoic acid FE}{Ethylene FE}*\frac{\# of electron transferred ethylene}{\# of electron transferred furoic aicd}*\frac{{MW}_{furoic acid}}{{MW}_{ethylene}}*{100}_{\frac{tonne ethylene}{day}}$$

$$Total fuoric acid production \left[ \frac{tonne}{day} \right]= Fuoric acid production from a_{HER}+ Fuoric acid production from Carnizzaro reaction$$

$$Fuoric acid production from Carnizzaro reaction=0.25*Fuoric acid production from anodic HER$$

With the reaction ratio between a-HER and the Cannizzaro reaction established at 2:1 based on experimental data at 400 mA/cm^2^, and considering that furfuryl alcohol solely arises from the Cannizzaro reaction, the calculation of total furfuryl alcohol production and furfural consumption is outlined below:

$$Total furfuryl alcohol production \left[ \frac{tonne}{day} \right]=0.25*Fuoric acid production from anodic HER*\frac{{MW}_{furfuryl alcohol}}{{MW}_{furoic acid}}$$

$$Total furfural consumption \left[ \frac{tonne}{day} \right]= Total fuoric acid production*\frac{{MW}_{furfural}}{{MW}_{furoic acid}}+Total furfuryl alcohol production*\frac{{MW}_{furfural}}{{MW}_{furfuryl alcohol}}$$

Therefore, the cost of furfural to make 100 tonne ethylene

$$Cost of furfural\left[ \frac{\$}{tonne ethylene} \right]= Total furfural consumption \left[ \frac{tonne}{day} \right]*Furfural cost \left[ \frac{\$}{tonne} \right]*\frac{1 day}{100 tonne ethylene}$$

As mentioned earlier, the synthesized furoic acid promptly undergoes deprotonation to form furoate under alkaline media. The precipitated furoate is subsequently separated and regenerated using sulfuric acid.^16^ To calculate the consumption of potassium hydroxide and the cost of sulfuric acid used for the regeneration process, the following equations are utilized:

$$Cost of KOH\left[ \frac{\$}{tonne ethylene} \right]=Total fuoric acid production*\frac{{MW}_{KOH}}{{MW}_{furoic acid}}*KOH cost \left[ \frac{\$}{tonne} \right]*\frac{1 day}{100 tonne ethylene}$$

$$Cost of H_{2}{SO}_{4}\left[ \frac{\$}{tonne ethylene} \right]=Total fuoric acid production*\frac{{MW}_{H_{2}{SO}_{4}}}{{MW}_{furoic acid}}*H_{2}{SO}_{4} cost \left[ \frac{\$}{tonne} \right]*\frac{1 day}{100 tonne ethylene}$$

4. Other operational costs

Other operating costs are calculated based on their proportional contributions to total capital expenditure (CAPEX), informed by recent literature and DOE modeling frameworks. Labor costs were estimated based on the requirement of approximately 10–15 full-time operators for a 100 tonne/day ethylene production facility, assuming a fully burdened wage of $50 / hour. This results in a labor-related operational cost equivalent to 9–13% of CAPEX.^16,17^ Routine maintenance, including repairs and the replacement of major components such as electrolyzer stacks, membranes, and catalysts, was assumed to require ~3% of CAPEX annually.^16,18^ Property taxes and insurance premiums were modeled as an annual expense corresponding to 2–3% of CAPEX under average assumptions, and up to 4–6% in conservative scenarios based on industrial benchmarks.^16,19^ Depreciation was treated as a straight-line charge distributed over a 20-year plant lifetime, corresponding to approximately 5% of CAPEX per year, consistent with U.S. DOE H2A protocols.^16,19,20^ In addition, general and administrative overheads, utility services, and consumable supplies were estimated to be approximately 30% of the total labor cost, reflecting standard multipliers used in electrochemical process design.^16,20^ The following equation summarizes the total operational costs as a function of CAPEX and labor.

$$Operational costs\left[ \frac{\$}{tonne ethylene} \right]=Labor costs +Maintenance costs+Taxes and Insurance costs+Depriciation costs+Miscellaneous overheads costs \left[ \frac{\$}{tonne ethylene} \right]$$

$$Labor costs\left[ \frac{\$}{tonne ethylene} \right]=CAPEX \left[ \frac{\$}{tonne ethylene} \right]*Labor factor$$

$$Maintenance costs\left[ \frac{\$}{tonne ethylene} \right]=Installation \left[ \frac{\$}{tonne ethylene} \right]*Maintenance factor$$

$$Taxes and Insurance costs\left[ \frac{\$}{tonne ethylene} \right]=CAPEX \left[ \frac{\$}{tonne ethylene} \right]*Taxes and insurance factor$$

$$Depriciation costs\left[ \frac{\$}{tonne ethylene} \right]=CAPEX \left[ \frac{\$}{tonne ethylene} \right]*Depriciation factor$$

$$Miscellaneous overheads costs\left[ \frac{\$}{tonne ethylene} \right]=Labor costs \left[ \frac{\$}{tonne ethylene} \right]*M.O. factor$$

$$CAPEX \left[ \frac{\$}{tonne ethylene} \right]=(Installation+Balance of Plant+PSA Capital cost+Distillation Capital costs)\left[ \frac{\$}{tonne ethylene} \right]$$

Following table indicates TEA values on COR & OER with parameter indicated in **Table S15** and **S16.**

**Table S15.** TEA values on COR & OER system for sensitivity analysis.

| Parameter | Optimistic | Average | Considerate |
| --- | --- | --- | --- |
| Costs ($/tonne ethylene) |  |  |  |
| Electrolyzer | 43 | 107 | 161 |
| Catalyst & Membrane | 5 | 14 | 27 |
| Electricity | 914 | 1218 | 1522 |
| Input chemical | 774 | 776 | 784 |
| Gas separation | 29 | 34 | 40 |
| Liquid separation | 55 | 66 | 77 |
| Installment | 35 | 63 | 90 |
| Balance of plant | 17 | 32 | 45 |
| Other operational costs | 43 | 104 | 180 |
| Total | 1969 | 2539 | 3114 |
| Potential benefit | 154 | -416 | -991 |

**Separate technoeconomic assessment on cathode and anode reaction**

Due to significant differences in the molecular weights of cathodic and anodic products, separate Techno-Economic Assessment (TEA) calculations were conducted, targeting a production of 100 tonnes of ethylene per day.

For the cathodic COR segment, plant-gate levelized costs were computed encompassing electrolyte, catalyst, membrane, electrolyzer, input chemical expenses, electricity, PSA gas separation costs (both capital and operating), distillation costs (both capital and operating), balance of plant, installation costs, and other operational expenditures.

For the anodic a-HER segment, a similar methodology was adopted as in the cathodic part, but with the inclusion of the costs of sulfuric acid and potassium hydroxide in the input chemical expenses and separation costs over furfural, furfuryl alcohol and furoic acid. In this calculation, we only considered costs and profits from anodic products such as hydrogen, furfural, furfuryl alcohol and furoic acid. Furthermore, since pure hydrogen gas flow is obtained at the anode outlet, PSA gas separation costs (both capital and operating) were excluded. Detailed values are provided in **Table S17**.

**Table S16.** TEA values on cathode COR reaction for sensitivity analysis.

| Parameter | Optimistic | Average | Considerate |
| --- | --- | --- | --- |
| Costs ($/tonne ethylene) |  |  |  |
| Electrolyzer | 16 | 39 | 59 |
| Catalyst & Membrane | 2 | 5 | 10 |
| Electricity | 335 | 447 | 558 |
| Input chemical | 773 | 776 | 783 |
| Gas separation | 29 | 34 | 40 |
| Liquid separation | 55 | 66 | 77 |
| Installment | 41 | 69 | 96 |
| Balance of plant | 20 | 35 | 48 |
| Other operational costs | 24 | 49 | 80 |
| Total | 1296 | 1520 | 1753 |
| Potential benefit | 826 | 602 | 370 |

**Table S17.** TEA values on anode a-HER reaction for sensitivity analysis.

| Parameter | Optimistic | Average | Considerate |
| --- | --- | --- | --- |
| Costs ($/tonne ethylene) |  |  |  |
| Electrolyzer | 16 | 39 | 59 |
| Catalyst & Membrane | 2 | 5 | 10 |
| Electricity | 335 | 447 | 558 |
| Input chemical | 97697 | 104779 | 114277 |
| Gas separation | - | - | - |
| Liquid separation | 898 | 1089 | 1272 |
| Installment | 58 | 88 | 117 |
| Balance of plant | 29 | 44 | 58 |
| Other operational costs | 55 | 100 | 156 |
| Total | 99090 | 106591 | 116507 |
| Potential benefit | 290019 | 282518 | 272601 |

Given the relatively small market size for furoic acid, estimated at 7.8 USD million,^16^ there are concerns regarding the accuracy of furoic acid market costs. To address this, we attempted to ascertain the furoic acid market price by plotting the furoic acid market price against potential profit/plant-gate levelized costs, utilizing our experimental results (**Figure S41**).

**Figure S41.** (a) Plot illustrating the ratio of potential profit to plant-gate levelized costs and (b) versus the assumed price of furoic acid.

Our analysis suggests that the system may achieve commercial viability at furoic acid market prices above approximately $1.7/kg, even under conservative assumptions. Previous reports have indicated a market price for furoic acid of around $6.23/kg.^15, 21^

7. **Supplementary Note 7**

**Downstream separation calculation with ASPEN simulation**

The downstream separation of the COR electrolyzer includes a cryogenic distillation purification step^1^ to remove all syngas from the gaseous outlet stream and obtain a 99.9wt% polymer-grade ethylene stream. AspenPlus v10 (AspenTech Inc., Bedford, MA) is used to model and design the purification process. Simulations are performed based on the gas outlet compositions of this work, demonstrating high concentrated ethylene stream, to assess the overall separation energy requirements to obtain a polymer grade ethylene. The design consists in a cryogenic cooler to bring the gaseous stream to temperature, followed by the distillation column to remove the syngas and obtain a liquid ethylene stream (-200°C).

**Figure S42.** Process Flow Diagram of the SOEC-COR-to-Ethylene downstream separation process.

The energy balance and fixed equipment parameters are displayed within the **Table S18** and **S19**. The energy duty presented here correspond to the requirements to achieve high purity ethylene in the experimental case of the **CuO & C-support_3 yielding 91% SPCE, producing a high-concentrated ethylene stream out of the electrolyzer (94.36wt%)**. The simulation data is exported directly from AspenPlus, thus the values are in cal/s. Afterwards, the energy duty is converted in GJ/ton ethylene based on the production flowrate and displayed in the **Table S18**.

**Table S18 -** AspenPlus equipment model data – parameters, conditions, and energy duty for distillation column with CuO & C-support_3.

| Name | DISTILL.1 |
| --- | --- |
| Property method | PENG-ROB |
| Number of stages | 20 |
| Condenser | PARTIAL-V |
| Reboiler | KETTLE |
| Number of phases | 2 |
| Top stage pressure (bar) | 1 |
| Condenser / top stage temperature (C) | -128.76 |
| Condenser / top stage pressure (bar) | 1 |
| Reboiler pressure (bar) | 1 |
| Reboiler temperature (C) | -117.47 |
| Condenser / top stage heat duty (cal/s) | -86970.79 |
| Reboiler heat duty (cal/s) | 165834.65 |

**Table S19.** AspenPlus equipment model data – parameters, conditions, and energy duty for heaters/coolers.

| Name | CRYOCOOL |
| --- | --- |
| Property method | PENG-ROB |
| Specified pressure (bar) | 1 |
| Specified temperature (C) | -200 |
| Calculated heat duty (cal/s) | -257080.8 |

**Table S20.** Separation energy requirements for COR-to-ethylene process - each experiment conducted within this work is listed below

| **Experimental system** | **Flow rate (sccm)** | **Current density (mA/cm^2^)** | **SPCE** | **Separation energy requirement (GJ/ton C_2_H_4_)** |
| --- | --- | --- | --- | --- |
| Control CuO | 20 | 200 | 4% | 19.52 |
| Control CuO | 1.12 | 200 | 69% | 2.74 |
| **CuO & C-support_3** | **0.82** | **200** | **91%** | **2.45** |
| CuO & C-support_3 | 1.15 | 300 | 94% | 2.19 |
| CuO & C-support_3 | 1.54 | 400 | 93% | 0.81 |
| CuO & C-support_3 | 20 | 200 | 4% | 18.68 |

8. **Supplementary Note 8**

**Extended stability data and component-level degradation analysis**

Extended stability experiments and post-stability diagnostics were used to separate AuCu anode behavior from device-level losses in the COR-a-HER membrane-electrode-assembly (MEA) cell. The analysis focuses on two observables: anode-side selectivity/structural retention and full-cell current density/impedance, which also include membrane, cathode, electrolyte, and transport effects.

In the furfural-based MEA operated potentiostatically at 0.8 V_cell_, maintaining stable current density beyond approximately 20 h was difficult, and the current density decreased to approximately 100 mA cm^-2^. The anode remained selective in this lower-current regime, with furoic acid and anode H_2_ Faradaic efficiencies of approximately 93-97%. Replacing the used membrane restored the current density from approximately 100 to 380 mA cm^-2^, whereas replacing the anode or cathode produced only a minor change. This behavior suggests that the main current loss in the furfural system is associated with the membrane/cell transport pathway rather than irreversible AuCu anode deactivation.

Photographs of the used anion-exchange membranes (AEMs), membrane-extract ^1^H NMR, and EIS provide additional support for this assignment (**Figure S42** and **S43**). The furfural-treated AEM shows brown discoloration, while the HCHO-treated AEM remains lighter in color. The furfural-treated membrane extract contains furan-derived resonances, and the furfural-treated cell shows a much larger low-frequency impedance than the HCHO-treated cell. These observations do not identify a single fouling species, but they are consistent with stronger retention/deposition of furan-derived molecules, possibly including oligomeric or polymeric residues, in the AEM. This furan-derived accumulation likely contributes to membrane/cell polarization during prolonged furfural operation^54,55^.

**Figure S42**. Photographs and 1H NMR analysis of species extracted from used AEMs after stability testing. 1H NMR spectra of DI-water extracts from used Sustainion X37-50 RT AEMs after MEA stability tests using (a) HCHO and (b) furfural as anodic reactants. The inset photographs show the corresponding AEMs after operation. The furfural-treated AEM shows brown discoloration and furan-derived NMR resonances, whereas the HCHO-treated AEM remains lighter in color and shows only weak non-diagnostic signals. These results are consistent with stronger retention of furan-derived species in the AEM after furfural operation.

**Formaldehyde control and extended formaldehyde-based stability test**

Formaldehyde was used as a non-furanic aldehyde control to test whether the AuCu anode can sustain a-HER operation when furan-derived membrane residues are minimized. Formaldehyde oxidation can proceed through analogous aldehyde-to-carboxylate oxidative dehydrogenation chemistry, but it lacks the furan ring that may promote retention or oligomer formation in the membrane. The lighter HCHO-treated AEM, weaker membrane-extract NMR signals, and smaller impedance increase after HCHO operation are therefore consistent with reduced furan-derived membrane/cell fouling, rather than complete elimination of all membrane effects^49-56^.

The COR-a-HER MEA was then operated at 0.8 V_cell_ using AuCu as the anode catalyst and formaldehyde as the anodic reactant. The cell maintained operation for more than 45 h before a temporary performance/selectivity loss associated with cathode-side salt accumulation. Cathode exchange restored the CO-reduction selectivity, indicating a reversible cathode-side contribution. After approximately 60 h, the cell was operated overnight without refreshing formaldehyde; the current density decreased but recovered after HCHO replenishment, consistent with aldehyde depletion rather than irreversible anode failure.

Time-resolved Faradaic efficiencies support this interpretation. The anode H2 FE remained 91-102%, and the anodic oxidation-product FE remained 92-102% over the measured time points. In contrast, the cathode ethylene FE temporarily decreased during the salt-accumulation period and recovered after cathode exchange. These data suggest that the AuCu anode remained highly selective for a-HER during the extended HCHO-based test, while the main temporary losses were related to cathode management or reactant supply^49-52^.

**Figure S43**. EIS analysis of AuCu before and after stability tests. Nyquist plots of AuCu measured before a-HER, after HCHO-based electrolysis, and after furfural-based electrolysis. The furfural-treated sample exhibits a much larger low-frequency impedance than the HCHO-treated sample, consistent with stronger transport/interfacial polarization after furfural operation.

**Table S21**. EIS metrics of AuCu before and after stability tests. The low-frequency impedance increases more strongly after furfural operation than after formaldehyde operation, consistent with larger interfacial/transport polarization in the furfural MEA.

| **Sample** | **Rs/HFR (ohm)** | **Re at 0.1 Hz (ohm)** | **-Im at 0.1 Hz (ohm)** | **\|Z\| at 0.1 Hz (ohm)** |
| --- | --- | --- | --- | --- |
| AuCu pre a-HER | 0.282 | 0.658 | 0.011 | 0.658 |
| AuCu after HCHO | 0.304 | 6.017 | 0.887 | 6.082 |
| AuCu after furfural | 0.448 | 36.93 | 89.11 | 96.46 |

**Figure S44**. Extended stability test of the AuCu-based MEA using formaldehyde as the anodic reactant. Current density-time profile of the COR-a-HER MEA operated potentiostatically at 0.8 V_cell_ with AuCu as the anode catalyst. The temporary performance/selectivity loss after approximately 45 h is attributed to cathode-side salt accumulation and is mitigated by cathode exchange. The later current decrease after overnight operation without HCHO refresh is reversed by HCHO replenishment, consistent with reactant depletion.

**Table S22**. Time-based Faradaic efficiencies during the extended formaldehyde-based stability test. The anodic liquid product is formate/formic acid. The temporary increase in cathode H_2_ FE and decrease in ethylene FE around 40-45 h are attributed to cathode-side salt accumulation; cathode exchange restores the original selectivity while the anode FE remains high.

| **Time (s)** | **Time (h)** | **Cathode H_2_ FE (%)** | **Ethylene FE (%)** | **Anode H_2_ FE (%)** | **Formate FE (%)** |
| --- | --- | --- | --- | --- | --- |
| 10500 | 2.9 | 13 | 54 | 97 | 95 |
| 25390 | 7.1 | 9 | 56 | 95 | 94 |
| 48246 | 13.4 | 10 | 53 | 96 | 97 |
| 68614 | 19.1 | 9 | 58 | 98 | 95 |
| 87417 | 24.3 | 16 | 55 | 99 | 96 |
| 118122 | 32.8 | 15 | 48 | 101 | 102 |
| 135975 | 37.8 | 20 | 45 | 93 | 95 |
| 145363 | 40.4 | 50 | 35 | 91 | 95 |
| 161176 | 44.8 | 65 | 28 | 95 | 96 |
| 174811 | 48.6 | 12 | 57 | 102 | 92 |
| 191267 | 53.1 | 7 | 55 | 96 | 94 |
| 203951 | 56.7 | 9 | 56 | 98 | 98 |
| 210685 | 58.5 | 11 | 53 | 96 | 95 |
| 255946 | 71.1 | 16 | 54 | 98 | 93 |

**Stoichiometry and Faradaic-efficiency basis for a-HER**

For FE accounting, we distinguish the formal two-electron aldehyde-to-carboxylate oxidation from the net a-HER stoichiometry. In alkaline electrolyte, the formal liquid-product half reaction is:

##

$$C_{5}H_{4}O_{2}+3{OH}^{-}\to C_{5}H_{3}O_{3}^{-}+2H_{2}O+{2e}^{-}$$

In the a-HER pathway, part of the reducing equivalent is released chemically as H2 at the anode. The corresponding net anodic stoichiometry is:

##

$$2R-CHO+4{OH}^{-}\to2R-COO^{-}+{H2}_{2}O+H_{2}+{2e}^{-}$$

Thus, each carboxylate product corresponds to one external electron and one half-equivalent of anodic H_2_ in the net a-HER stoichiometry. Mechanistically, aldehyde hydration/gem-diol formation and C-H cleavage generate surface H* intermediates on Cu-based sites; neighboring H* species can recombine and desorb as H_2_. The anode H_2_ FE is measured by gas chromatography, and the liquid product FE is measured by NMR. Because furfural can also undergo non-Faradaic Cannizzaro chemistry, H_2_ FE and liquid-product FE are reported together to evaluate a-HER selectivity^49-53,56^.

**Overall stability interpretation**

Overall, the data suggest that AuCu remains an active and selective a-HER anode under the tested conditions, whereas the severe current decay in the furfural-based MEA is more likely associated with membrane/cell transport losses. The furfural case shows current recovery after membrane replacement, furan-derived NMR signatures in the used AEM, visible AEM discoloration, and a large increase in low-frequency impedance. The formaldehyde control reduces this furan-specific pathway and shows extended operation, although temporary cathode salt accumulation and aldehyde depletion still require cell-management strategies^49-56^.

These findings point to practical routes for improving long-term furfural-based COR-a-HER operation. Possible strategies include using more chemically inert porous separators, such as PTFE-based separators, when product-separation requirements allow; exploring bipolar membranes or other architectures that better control ion transport and local pH; and developing a-HER catalysts that operate at milder or near-neutral pH to reduce alkaline furfural polymerization/oligomerization and membrane fouling. These are proposed engineering directions rather than outcomes demonstrated in the present work.

**References**

1. Biesinger, M.C et al. Resolving surface chemical states in XPS analysis of first row transition metals, oxides and hydroxides: Sc, Ti, V, Cu and Zn. *Smart, Appl. Surf. Sci.* **257,** 887-898 (2010).
2. Imamov, R.M. et al. *Physica Status Solidi, Sectio A: Applied Research* **56**, 127 - 131, (1956).
3. Kern, R. et al. *Golden Book of Phase Transitions* **1**, 1 - 123, (2002).
4. Tokola, E. et al. *Annales Academiae Scientiarum Fennicae, Series A6: Physica* **223**, 1 - 10, (1967).
5. Waseda, Y. et al. *Journal of Materials Science* **23**, 757 - 760, (1988).
6. Liang C.K. et al. *Wu Li Hsueh Pao Acta Physica Sinica* **22**, 669 - 697, (1966).
7. Ozden, A. et al. Cascade CO_2_ electroreduction enables efficient carbonate-free production of ethylene. *Joule* **5**, 1–14 (2021).
8. Ozden, A. et al. Energy- and carbon-efficient CO_2_/CO electrolysis to multicarbon products via asymmetric ion migration-adsorption. *Nat. Energy* **8**, 179–190 (2023).
9. Ripatti, D.S. et al*.* Carbon Monoxide Gas Diffusion Electrolysis that Produces Concentrated C2 Products with High Single-Pass Conversion (vol 3, pg 240, 2019). *Joule* **3**, 2581-2581 (2019).
10. Yadegari, H. et al. Glycerol Oxidation Pairs with Carbon Monoxide Reduction for Low-Voltage Generation of C2 and C3 Product Streams. *Acs Energy Letters* **6**, 3538-3544 (2021).
11. Wu, H. et al. Selective and energy-efficient electrosynthesis of ethylene from CO_2_ by tuning the valence of Cu catalysts through aryl diazonium functionalization *Nat. Energy* **9**, 422-433 (2024).
12. Jouny, M. et al. General Techno-Economic Analysis of CO_2_ Electrolysis Systems. *Ind. Eng. Chem. Res.* **59**, 8121-8123 (2020).
13. Park, S. et al*.* High carbon efficiency in CO-to-alcohol electroreduction using a CO reservoir. *Joule* **7**, 2335-2348 (2023).
14. Wang, X. et al. Efficient electrosynthesis of n-propanol from carbon monoxide using a Ag–Ru–Cu catalyst. *Nat. Energy.* **7**, 170-176 (2022).
15. Na, J. et al. General technoeconomic analysis for electrochemical coproduction coupling carbon dioxide reduction with organic oxidation. *Nat. Comm.* **10**, 5193 (2019).
16. U.S. Department of Energy, H2A Hydrogen Analysis Model Documentation. <https://www.hydrogen.energy.gov/h2a_analysis.html>
17. Bhandari, R. et al., Life cycle assessment of hydrogen production via electrolysis – a review. *Journal of cleaner production* **8**, 151–163 (2014).
18. E. S. Rubin et al. A proposed methodology for CO_2_ capture and storage cost estimates *Int. J. Greenh. Gas Control*, **17**, 488 (2013).
19. Z. Zishuai et al. Conversion of reactive carbon solutions into CO at low voltage and high carbon efficiency. *ACS Cent Sci*, **8**, 749-755 (2022).
20. M. S. Peters et al., “Plant Design and Economics for Chemical Engineers,” 5th Ed. (2003).
21. Ghatta, A. et al. Sustainable and efficient production of furoic acid from furfural through amine assisted oxidation with hydrogen peroxide and its implementation for the synthesis of alkyl furoate. *RSC Sustain.* **1**, 303-309 (2023).
22. Paturska, A. et al. Economic assessment of biomethane supply system based on natural gas infrastructure. *Energy Procedia* **72**, 71-78 (2015).
23. Yang. F. et al. Dynamics of bulk and surface oxide evolution in copper foams for electrochemical CO_2_ reduction. *Communications Chemistry*. **7**, 66, (2024).
24. Jiang. S. et al. New aspects of operando Raman spectroscopy applied to electrochemical CO_2_ reduction on Cu foams. *J. Chem. Phys*. **150**, 041718, (2019).
25. Jiang. S. et al. Copper carbonate hydroxide as precursor of Interfacial CO in CO_2_ electroreduction. *ChemSusChem*. **15**, e20212506, (2022).
26. Deng, Y. et al. In situ Raman spectroscopy of copper and copper oxide surfaces during electrochemical oxygen evolution reaction: identification of Cu(III) oxides as catalytically active species. *ACS. Catalysis*. **6**, 2473-2481 (2016).
27. Moradzaman. M. et al. In Situ Raman Study of potential-dependent surface adsorbed carbonate, CO, OH, and C species on Cu electrodes during electrochemical reduction of CO_2_. *ChemElectroChem*. **8**, 1478-1485, (2021).
28. Cavalca. F. et al. Nature and distribution of stable subsurface oxygen in copper electrodes during electrochemical CO_2_ reduction. *The Journal of Physical Chemistry C.* **121**, 45, (2017).
29. Ostervold. L. et al. Identifying the local atomic environment of the “Cu^3+^” state in alkaline electrochemical systems. *ACS Appl. Mater. Interfaces*. **15**, 27878-27892, (2023).
30. Okada. M. et al. Experimental and Theoretical Studies on Oxidation of Cu-Au Alloy Surfaces: Effect of Bulk Au Concentration. *Scientific Reports*. **6**, 31101, (2016).
31. Jiao. S. et al. Descriptors for the evaluation of electrocatalytic reactions: d-band theory and beyond. *Adv. Funct. Mater*. **32**, 2107651, (2022).
32. Sham. T.K. et al. Observations on the d-band width of AuAg and AuCu alloys. *Solid State Commun*. **80**, 29–32, (1991).
33. Liu, Z. et al. The Effect of the Reference Electrode Position on the Measurement of Half Cell Polarization in Proton-Exchange Membrane Fuel Cells. *J. Electrochem. Soc.* **159**, F554–F559 (2012).
34. Dedigama, I. et al. Stable Reference Electrode in Polymer Electrolyte Membrane Electrolyser for Three-Electrode Measurements. *J. Power Sources* **265**, 97–103 (2014).
35. Inaba, M. et al. An experimental study on the placement of reference electrodes in alkaline polymer electrolyte membrane fuel cells. *J. Power Sources* **353**, 19–27 (2017).
36. Kresse, G. et al. Ab initio molecular dynamics for liquid metals. *Phys. Rev. B* **47**, 558–561 (1993).
37. Kresse, G. et al. Efficient iterative schemes for ab initio total-energy calculations using a plane-wave basis set. *Phys. Rev. B* **54**, 11169–11186 (1996).
38. Perdew, J. P. et al. Generalized gradient approximation made simple. *Phys. Rev. Lett.* **77**, 3865–3868 (1996).
39. Grimme, S. et al. Effect of the damping function in dispersion corrected density functional theory. *J. Comput. Chem.* **32**, 1456–1465 (2011).
40. Grimme, S. et al. A consistent and accurate ab initio parametrization of density functional dispersion correction (DFT-D) for the 94 elements H–Pu. *J. Chem. Phys.* **132**, 154104 (2010).
41. Larsen, A. H. *et al. The Atomic Simulation Environment – a Python library for working with atoms.* *J. Phys. Condens. Matter* **29**, 273002 (2017).
42. Wang, V. et al. VASPKIT: A user-friendly interface facilitating high-throughput computing and analysis using VASP code. *Comput. Phys. Commun.* **267**, 108033 (2021).
43. Islam, S. M. R. et al. An implicit electrolyte model for plane-wave density functional theory exhibiting nonlinear response and a nonlocal cavity definition. *J. Chem. Phys.* **159**, (2023).
44. Nosé, *S. et al. A molecular dynamics method for simulations in the canonical ensemble.* *Mol. Phys.* **100**, 191–198 (2002).
45. Hammer, B. et al. Theoretical surface science and catalysis—calculations and concepts. *Adv. Catal.* **45**, 71–129 (2000).
46. Nørskov, J. K. *et al.* Fundamental Concepts in Heterogeneous Catalysis. (John Wiley & Sons, 2014).
47. Liu, Y.; Hight Walker, A. R. Preferential Outward Diffusion of Cu during Unconventional Galvanic Replacement Reactions between HAuCl4 and Surface-Limited Cu Nanocrystals. *ACS Nano* **5**, 6843-6854 (2011).
48. Cheng, H.; Wang, C.; Qin, D.; Xia, Y. Galvanic Replacement Synthesis of Metal Nanostructures: Bridging the Gap between Chemical and Electrochemical Approaches. *Acc. Chem. Res*. **56**, 900-909 (2023).
49. Wang, T. et al. Combined anodic and cathodic hydrogen production from aldehyde oxidation and hydrogen evolution reaction. *Nat. Catal*. **5**, 66-73 (2022).
50. Liu, H. et al. Ultra-low voltage bipolar hydrogen production from biomass-derived aldehydes and water in membrane-less electrolyzers. *Energy Environ. Sci*. **15**, 4175-4189 (2022).
51. Pan, Y. et al. Unveiling the synergistic effect of multi-valence Cu species to promote formaldehyde oxidation for anodic hydrogen production. *Chem* **9**, 963-977 (2023).
52. Ramos, N. C.; Holewinski, A. Recent advances in anodic hydrogen production: Electrochemical oxidative dehydrogenation of aldehydes to carboxylates. *Curr. Opin. Electrochem*. **45**, 101484 (2024).
53. Kikhtyanin, O.; Lesnik, E.; Kubicka, D. The occurrence of Cannizzaro reaction over Mg-Al hydrotalcites. *Appl. Catal. A Gen*. **525**, 215-225 (2016).
54. Salvatore, D. A. et al. Designing anion exchange membranes for CO2 electrolysers. *Nat. Energy* **6**, 339-348 (2021).
55. Sassenburg, M.; Kelly, M.; Subramanian, S.; Smith, W. A.; Burdyny, T. Zero-Gap Electrochemical CO2 Reduction Cells: Challenges and Operational Strategies for Prevention of Salt Precipitation. *ACS Energy Lett*. **8**, 321-331 (2023).
56. Yang, M. et al. Correlating the Valence State with the Adsorption Behavior of a Cu-Based Electrocatalyst for Furfural Oxidation with Anodic Hydrogen Production Reaction. *Adv. Mater*. **35**, 2304203 (2023).
57. Ren, W.; Ma, W.; Hu, X. Tailored water and hydroxide transport at a quasi-two-phase interface of membrane electrode assembly electrolyzer for CO electroreduction. *Joule* **7**, 2349-2360 (2023).
58. Jin, J. et al. Constrained C2 adsorbate orientation enables CO-to-acetate electroreduction. *Nature* **617**, 724-729 (2023).
59. Dorakhan, R. et al. A silver-copper oxide catalyst for acetate electrosynthesis from carbon monoxide. *Nature Synthesis* **2**, 448-457 (2023).
60. Jouny, M.; Luc, W.; Jiao, F. High-rate electroreduction of carbon monoxide to multi-carbon products. *Nature Catalysis* **1**, 748-755 (2018).
61. Miao, R. K. et al. CO electrolysers with 51% energy efficiency towards C2+ using porous separators. *Nature Energy* **10**, 1197-1204 (2025).
62. Xie, K.; Ozden, A.; Miao, R. K.; Li, Y.; Sinton, D.; Sargent, E. H. Eliminating the need for anodic gas separation in CO2 electroreduction systems via liquid-to-liquid anodic upgrading. *Nature Communications* **13**, 3070 (2022).
